# Evolution of the clinical-stage hyperactive TcBuster transposase as a platform for robust non-viral production of adoptive cellular therapies

**DOI:** 10.1016/j.ymthe.2024.04.024

**Published:** 2024-04-16

**Authors:** Joseph G. Skeate, Emily J. Pomeroy, Nicholas J. Slipek, Bryan J. Jones, Bryce J. Wick, Jae-Woong Chang, Walker S. Lahr, Erin M. Stelljes, Xiaobai Patrinostro, Blake Barnes, Trevor Zarecki, Joshua B. Krueger, Jacob E. Bridge, Gabrielle M. Robbins, Madeline D. McCormick, John R. Leerar, Kari T. Wenzel, Kathlyn M. Hornberger, Kirsti Walker, Dalton Smedley, David A. Largaespada, Neil Otto, Beau R. Webber, Branden S. Moriarity

**Affiliations:** 1 Department of Pediatrics, University of Minnesota, Minneapolis, MN 55455, USA; 2 Masonic Cancer Center, University of Minnesota, Minneapolis, MN 55455, USA; 3 Center for Genome Engineering, University of Minnesota, Minneapolis, MN 55455, USA; 4 Bio-Techne, Minneapolis, MN 55413, USA

**Keywords:** cellular therapy engineering, immunotherapy, transposons, NK cells, T cell, chimeric antigen receptors, engineering pipeline, high-throughput mutant screening

## Abstract

Cellular therapies for the treatment of human diseases, such as chimeric antigen receptor (CAR) T and natural killer (NK) cells have shown remarkable clinical efficacy in treating hematological malignancies; however, current methods mainly utilize viral vectors that are limited by their cargo size capacities, high cost, and long timelines for production of clinical reagent. Delivery of genetic cargo via DNA transposon engineering is a more timely and cost-effective approach, yet has been held back by less efficient integration rates. Here, we report the development of a novel hyperactive TcBuster (TcB-M) transposase engineered through structure-guided and *in vitro* evolution approaches that achieves high-efficiency integration of large, multicistronic CAR-expression cassettes in primary human cells. Our proof-of-principle TcB-M engineering of CAR-NK and CAR-T cells shows low integrated vector copy number, a safe insertion site profile, robust *in vitro* function, and improves survival in a Burkitt lymphoma xenograft model *in vivo*. Overall, TcB-M is a versatile, safe, efficient and open-source option for the rapid manufacture and preclinical testing of primary human immune cell therapies through delivery of multicistronic large cargo via transposition.

## Graphical abstract

**Figure:**
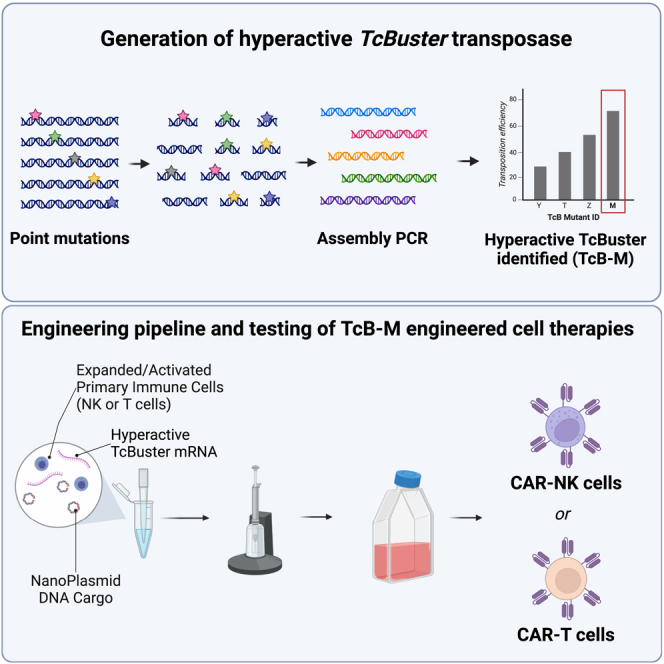

## Teaser

Genome engineering via DNA transposons is a cost-effective, non-viral approach to produce cellular therapies. Here, Moriarity and colleagues report a novel structure-guided and *in vitro* evolution approach to generating hyperactive TcBuster (TcB-M) transposase and provide a detailed pipeline for engineering primary human NK and T cells for cellular therapies.

## Introduction

DNA transposons are natural DNA transfer vehicles that can be used for stable DNA integration into genomes. In nature, they exist as well-defined elements in which the transposase gene is flanked by inverted terminal repeats (ITRs) that encode transposase binding sites (reviewed in Muñoz-López and García-Pérez^1^). Genetic engineering using transposons is accomplished by flanking an expression cassette with ITRs and co-delivering the transposase enzyme via an expression plasmid, mRNA, or protein.^2^ Several DNA transposon systems have been developed in such a manner and used in mammalian cell engineering. In the 1990s, the *Sleeping Beauty* (*SB*) transposon system was molecularly reconstructed by eliminating inactivating mutations found in members of the Tc1/mariner family of transposons isolated from fish, and has since been used for stable gene transfer and insertional mutagenesis in many vertebrate cell types, including human cells.^3^^,^^4^^,^^5^ Subsequently, the *piggyBac* (*PB*) and *Tol2* transposable elements were isolated from insects and fish, respectively, and have since been engineered for enhanced activity in mammalian cells.^6^^,^^7^ Collectively, *SB*, *PB*, and *Tol2* can all be used as non-viral tools for stable gene delivery, and each of these systems has been used for gene delivery in primary human lymphocytes.^8^

Transposons have many meaningful advantages as an alternative to viral vectors for stable gene transfer. Several clinical gene therapy products have been developed using CD34+ hematopoietic stem cells or T cells genetically modified using recombinant viruses; namely γ-retroviruses, and lentiviruses.^9^^,^^10^^,^^11^^,^^12^^,^^13^^,^^14^^,^^15^^,^^16^^,^^17^^,^^18^^,^^19^ These delivery methods carry a heightened risk of insertional mutagenesis via activation of proto-oncogenes or inactivation of tumor-suppressor genes.^20^^,^^21^^,^^22^ A comparative study of the target site-integration properties of hyperactive forms of *SB* and *PB* transposons as well as gammaretroviral and lentiviral systems in primary human CD4+ T cells ranked their safety profiles based on multiple criteria, including distance from the 5′-end of any gene and distance from any cancer-related gene. This analysis established *SB* as having the most favorable integration profile, followed by *PB*, suggesting that engineering via transposition is a safer alternative to engineering with viral vectors.^23^ Yet, any vector that integrates into chromosomes poses the risk of insertional mutagenesis. In addition, large-scale manufacturing of these viral vectors for clinical use is costly, limited in cargo size, time-consuming, and impedes progression through clinical trials; particularly for academic institutions and emerging biotech companies. Thus, the use of transposon systems has been pursued as an alternative to viral vectors due to rapid, reproducible, cost-effective production, and a more favorable safety profile.^10^^,^^20^^,^^24^

A novel representative of the hAT superfamily of transposons is *TcBuster*, originally isolated from the red flour beetle.^25^ *TcBuster* has been shown to be active in human cell lines with a comparable transposition efficiency to the originally described *SB* and *PB*; however*,* no study to date has performed a comprehensive optimization of the TcBuster transposase enzyme to enhance its activity, as has been done for SB and PB.^26^^,^^27^^,^^28^^,^^29^^,^^30^^,^^31^^,^^32^ Here, we describe the development of hyperactive *TcBuster* using a high-throughput screen of 3 million generated variants and demonstrate its application to engineering primary chimeric antigen receptor (CAR) natural killer (NK) and CAR-T cells. With our optimized engineering process, we achieved manufacturing of enriched (>99%+) CD19 CAR-expressing NK and T cells and demonstrate functional efficacy both *in vitro* and *in vivo*. Our work provides a versatile, cost-effective, open-source, and clinically scalable platform for the non-viral manufacture of engineered cellular therapies for treatment of cancer, autoimmune disease, and beyond.

## Results

### Directed evolution of a hyperactive *TcBuster* transposase

**Figure 1:**
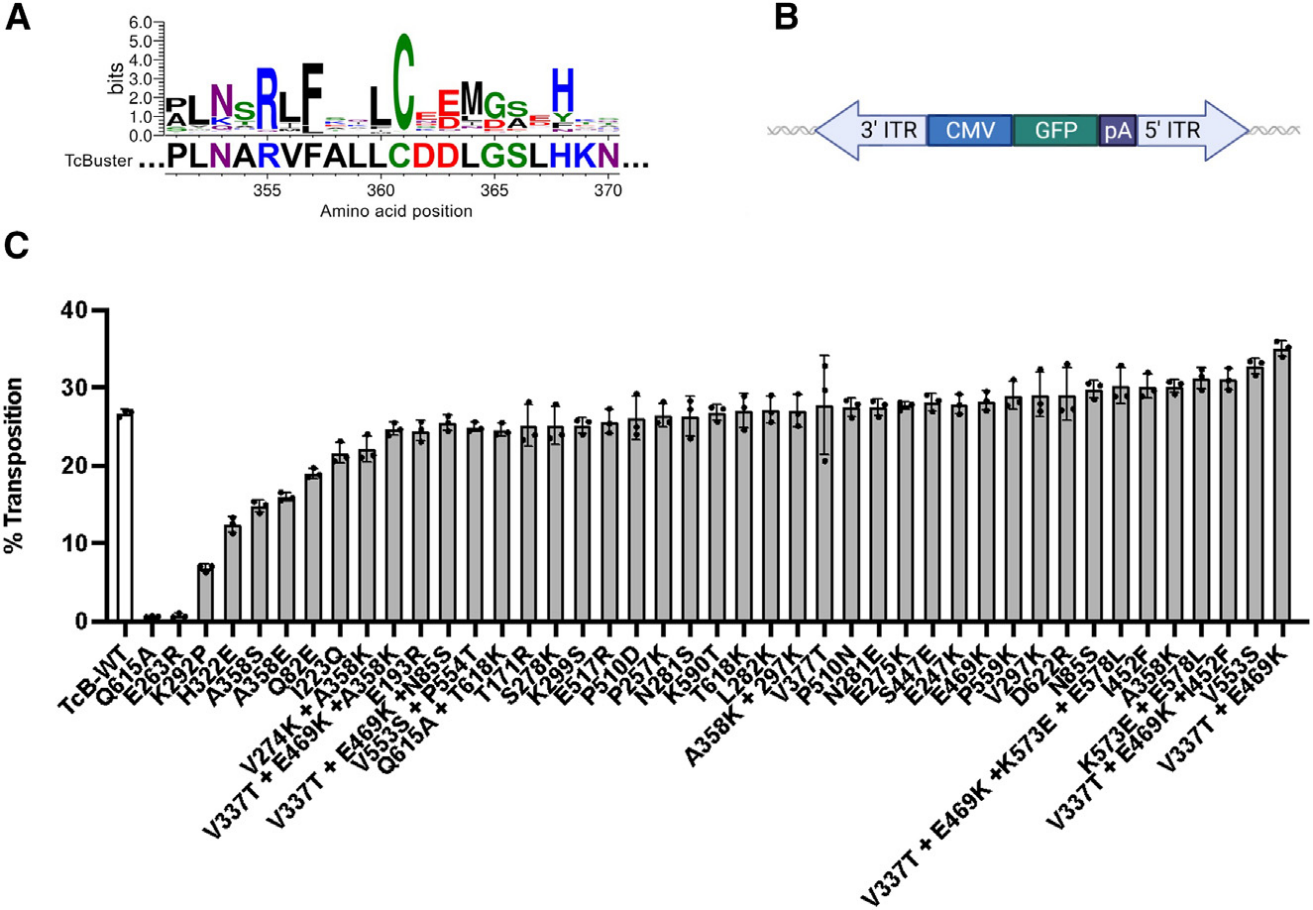
Identification of conserved amino acids within hAT family transposase provides hyperactive substitution candidates for *TcBuster* (A) A web logo plot of conserved amino acids across hAT family transposase members compared with *TcBuster*. Candidate hyperactive substitutions are conserved amino acids not found in TcBuster. V356L and L364M depicted here are candidate hyperactive substitutions. (B) A GFP transgene being driven by a CMV promoter within *TcBuster* Inverted Tandem Repeats. (C) HeLa cells were transiently co-transfected with a *TcBuster* variant and GFP transposon. Ten days following transfection, cells were measured for stable GFP expression as a measure of transposition efficiency (*n* = 3, SD shown).

*TcBuster* has been shown to be active in human cells, albeit at very low efficiencies.^27^ As demonstrated with the *Sleeping Beauty (SB)* and *PiggyBac (PB)* transposon systems, the activity of DNA transposase enzymes can be enhanced by incorporating phylogenetically conserved amino acids, structure-guided mutagenesis, and gene-shuffling approaches.^31^^,^^32^ Therefore, we reasoned that we could deploy these approaches to construct a hyperactive *TcBuster* system. We first identified 108 mutations using a combination of consensus finder and manual selection of common mutations from close homologs that could be substituted in *TcBuster* transposase to increase its enzymatic activity (Figure 1A).^33^ To create a *TcBuster* transposon for measuring transposition activity, we used the inverted terminal repeats (ITRs) and consensus target duplication sites (TSDs) previously published and incorporated a eGFP cDNA driven by the CMV promoter (Figure 1B).^26^^,^^27^ In HeLa cells, a handful of these single amino acid substitutions only slightly improved the activity of *TcBuster* in initial screens and only the combination of V377T and E469K yielded noticeable additive effects (Figure 1C). Further combinations had negative or no effect on enzyme activity. Finding mutations that work well in many contexts would thus be more amenable to combining with other beneficial mutations, necessitating a random combinatorial library of these mutations. Considering the large number of combinations from 108 individual substitutions (7,558 possible double-mutations) and combinations from all of these individual mutations (>10^32^), it was necessary to develop a high-throughput DNA shuffling strategy and screen in mammalian cells.

For this approach, a library of mutant transposases was created by shuffling the (108) amino acid substitutions via DNAse treatment and primerless assembly PCR.^34^^,^^35^ To identify additive and synergistic combinations, we created an enzyme library of two to three substitutions per cDNA.^31^ The quality of the library was determined by performing PacBio Circular Consensus Sequencing (CCS) sequencing, where we determined the average number of mutations per cDNA was 4.4 (Figure S1). A complete library with all combinations up to four mutations is >5 million *TcBuster* variants.

**Figure 2:**
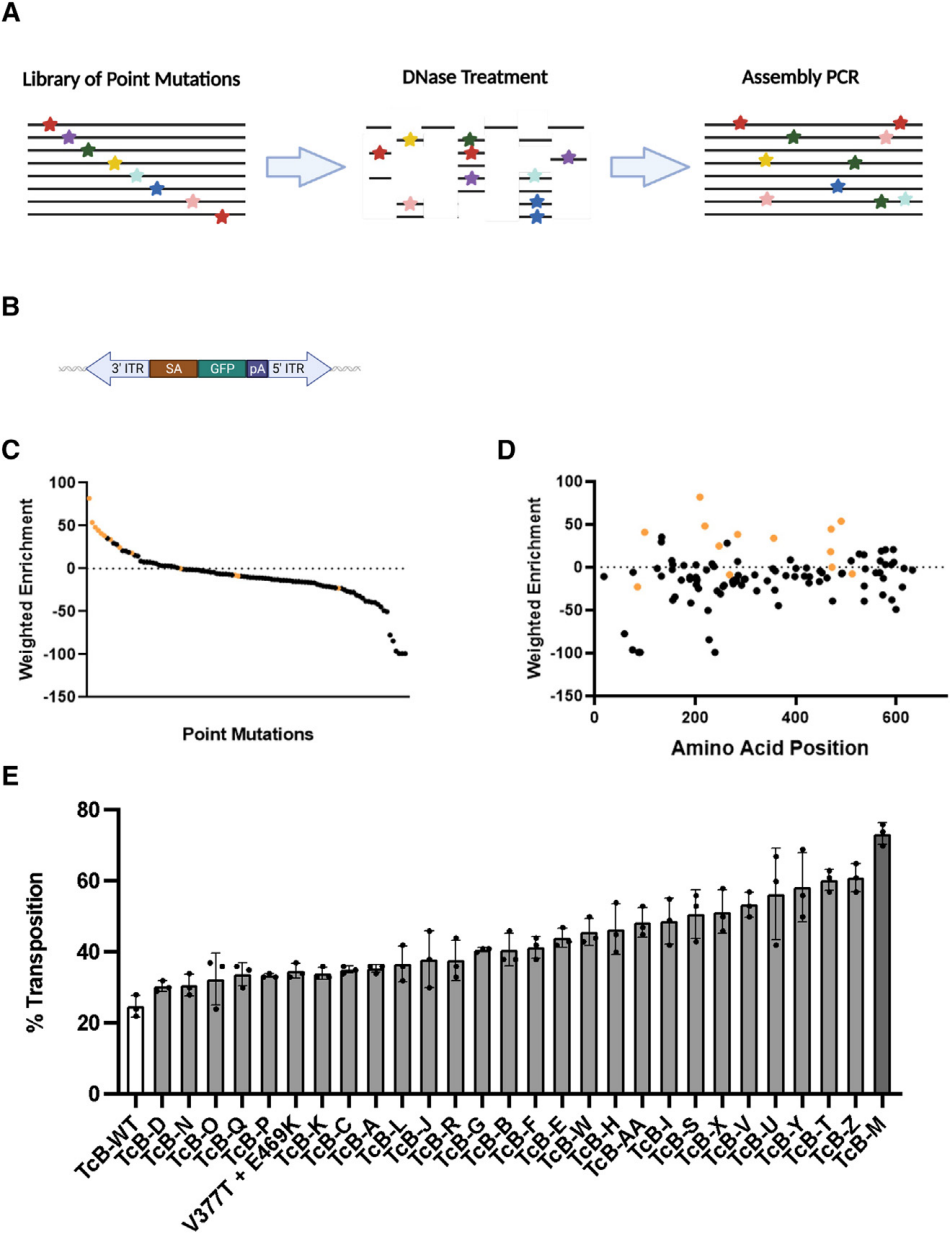
High-throughput screening platform for the creation of hyperactive *TcBuster* (TcB-M) (A) Workflow for the creation of a diverse TcBuster variant library. The 108 curated mutations were introduced as single point mutations into TcBuster. The single point mutants were DNase I digested and then subjected to primerless PCR, leading to the shuffling of the point mutations and an average of 4.4 mutations per variant. (B) An example splice acceptor transposon used for hyperactive *TcBuster* variant screening to limit the amount of episomal expression. (C) A plot of all point mutations in the *TcBuster* library and their weighted enrichment. Orange indicating the mutations found in TcB-M. (D) A plot of all point mutations in the *TcBuster* library weighted enrichment relative to their position in the TcBuster protein. (E) *TcBuster* variants were made by making various combinations of enriched mutations from the hyperactive screen. HeLa cells were transiently co-transfected with a *TcBuster* variants and GFP transposon. Ten days following transfection, cells were measured for stable GFP expression as a measure of transposition efficiency (*n* = 3, SD shown).

We then performed a bulk population-based high-throughput screen in mammalian cells (Figure 2A), allowing us to examine a library of 3 million unique TcBuster variants. We generated a lentiviral library and transduced HeLa cells at an MOI 0.3 to ensure that most cells received 0 to 1 copies of a *TcBuster* variant based on Poisson distribution.^36^ For screening, we reasoned that hyperactive enzymes would have the highest enzymatic activity, i.e., transposition events, with little substrate (transposon) in the shortest amount of time. Thus, we transfected HeLa cells stably expressing the *TcBuster* transposase library with three transposons containing hygromycin, GFP, or mCherry, and selected for cells that had all three transposons stably integrated. To limit the amount of substrate, we transfected a higher (500 ng) and lower (50 ng) amount of each transposon into the HeLa cell population carrying the *TcBuster* library. In order to avoid episomal expression, we deployed a splice acceptor to drive the expression of the transgenes rather than a constitutive promoter (Figure 2B). This approach also likely further enriches hyperactive mutants as many insertions will not integrate near active genes that can express the transposon transgene through splicing.

Following transposition, cells were selected with hygromycin and subsequently FACS sorted for bright (many transgene copies) and double-positive (fewer copies) GFP/mCherry double positivity (Figure 2B and Figure S1). NGS analysis of bright GFP+/mCherry+ cells revealed 19 positively enriched amino acid substitutions for cells transfected with low amounts of transposon and 31 for cells transfected with high amounts of transposon (Figures 2C and 2D). Interestingly, most mutations were enriched when using high or low amounts of transposon, regardless of double-positive or bright populations. Mutations from cells transfected with low amounts of transposon were more highly enriched or severely depleted vs. cells transfected with high amounts of transposon, likely in part because far fewer enzyme variants were represented in the low transposon screen.

Next, the most enriched transposase variants were manually combined and tested for activity in HeLa cells (Figure 2E). The *TcBuster* variant, TcB-M, that performed the best contained 14 amino acid substitutions, which included the originally identified hyperactive mutant (V377T, E469K).

### Optimized engineering of CAR-NK cells using hyperactive *TcBuster*

**Figure 3:**
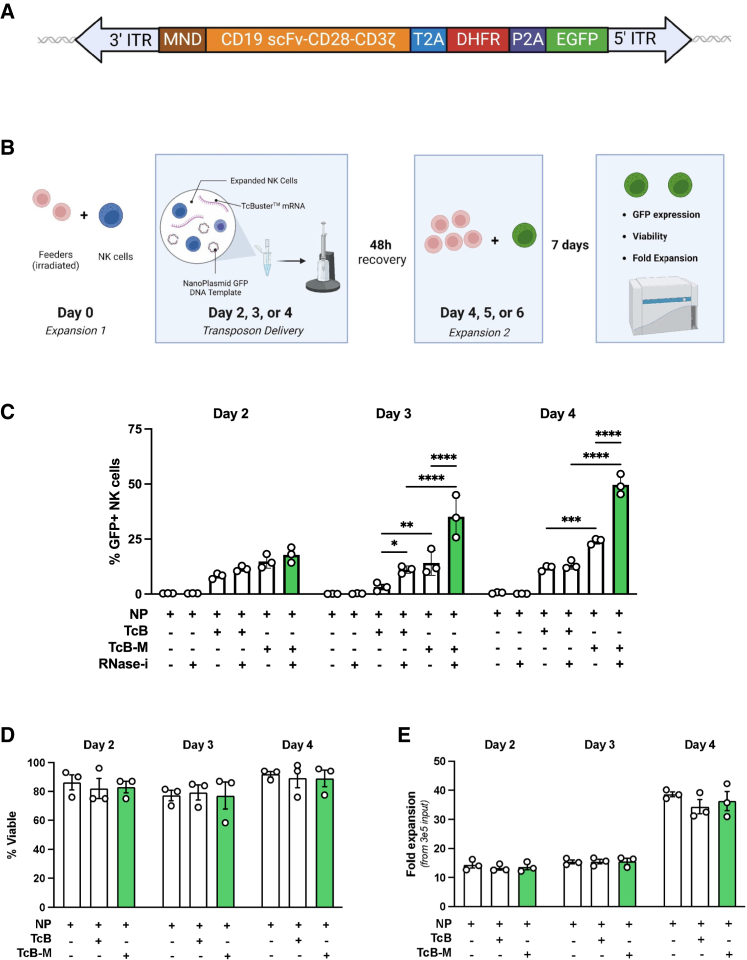
Delivery of a CD19-CAR-DHFR-eGFP transposon to NK cells and optimized engineering pipeline incorporating MTX selection using the *TcBuster* Transposon System for CAR-NK production (A) The 4.27-kb transposon flanked by *TcBuster* ITRs, containing an MND promoter, second-generation CD19 CAR, methotrexate-resistant DHFR mutant, and enhanced GFP. Elements are separated by 2A ribosomal skip sequences. This cargo was cloned into a nanoplasmid (NP) backbone for delivery. (B–E) Primary human peripheral blood leukocytes (PBL) NK cells (*n* = 3 human donors) were expanded for 2, 3, or 4 days with mbIL21-and 41BBL-expressing K562 feeder cells at a 2:1 (feeder:NK) ratio. NK cells were electroporated with the nanoplasmid transposon (NP) alone or in combination with mRNA encoding either *TcBuster* (TcB) or the hyperactive mutant *TcBuster* (TcB-M). Two days after electroporation, NK cells were expanded with feeder cells (5:1 feeder:NK ratio) for 1 week to allow for the loss of transient NP expression. After expansion, GFP expression was measured by flow cytometry (C), viability was measured by trypan blue exclusion (D), and fold expansion was calculated from input numbers (E) (SD shown, ns = not significant, ∗*p* < 0.05, ∗∗*p* < 0.01, ∗∗∗*p* < 0.001, ∗∗∗∗*p* < 0.0001).

We then deployed TcB-M to create a robust platform for the manufacture and enrichment of CAR-expressing NK cells from primary human peripheral blood leukocytes (PBL) NK cells. Using both the original version of TcB and TcB-M, we optimized transposon engineering of PBL NK with a CD19-CAR-DHFR-eGFP transposon expression cassette (4.37 kb transposon, Figure 3A). Our previously developed methods for efficient RNA delivery of CRISPR-Cas9 to primary human PBL NK cells were utilized as a starting point for further optimization.^37^ Specifically, we previously stimulated NK cells by coculture with irradiated membrane-bound interleukin (IL)21 (mbIL21)- and 41BBL-expressing K562 feeder cells for 7 days before electroporating them with mRNA encoding Cas9 and chemically modified single guide RNA.^37^ Unfortunately, using this process to deliver transposon plasmid and mRNA encoding the original *TcBuster* transposase resulted in low integration efficiency as measured by GFP expression (2.07% ± 0.37%) and poor recovery (12.00% ± 0.58%) of electroporated cells (Figures S2A and S2B). Based on our engineering experience with other cell types, we hypothesized that delivering DNA cargo earlier in the activation process may result in better engineering outcomes.^38^ To test this, we electroporated NK cells with transposon plasmid and the original *TcBuster* (TcB) or the hyperactive *TcBuster* mutant transposase (TcB-M) on day 2, 3, or 4 of feeder cell-mediated activation (Figure 3A). In line with our hypothesis, we observed higher transposition efficiency at earlier time points, with the highest efficiency on day 4 of activation with significant differences seen between TcB and TcB-M (11.85% ± 1.26% for TcB and 24.05% ± 1.36% for TcB-M) (Figure 3C).

The activation of NK cells is known to lead to the upregulation of ribonucleases.^39^ As the transposase is delivered as mRNA and would be subject to rapid degradation in the presence of high levels of these enzymes, we tested the treatment of NK cells with a pan ribonuclease inhibitor (RNase A, RNase B, and RNase T2) for 5 min prior to electroporation with transposase mRNA (Figures 3C–3E). We found that the addition of the RNase inhibitor enhanced transposition efficiency significantly, and again observed the highest transposition efficiency when electroporation was performed on day 4 of NK cell activation (Figure 3C, 13.23% ± 1.89% for TcB and 49.63% ± 4.64% for TcB-M). We then re-expanded the NK cells 48-h post-electroporation for 1 week and compared viability and fold expansion for each electroporation time point. While differences in cell viability were minimal (Figure 3D), NK cells electroporated on day 4 of activation had significantly higher fold expansion than those electroporated on day 2 or 3 (Figure 3E). Thus, optimal stable transposition in NK cells was achieved by electroporation on day 4 of expansion, pre-treatment with RNase inhibitor, and the use of TcB-M transposase.

### Enrichment of CAR-NK cells using methotrexate

**Figure 4:**
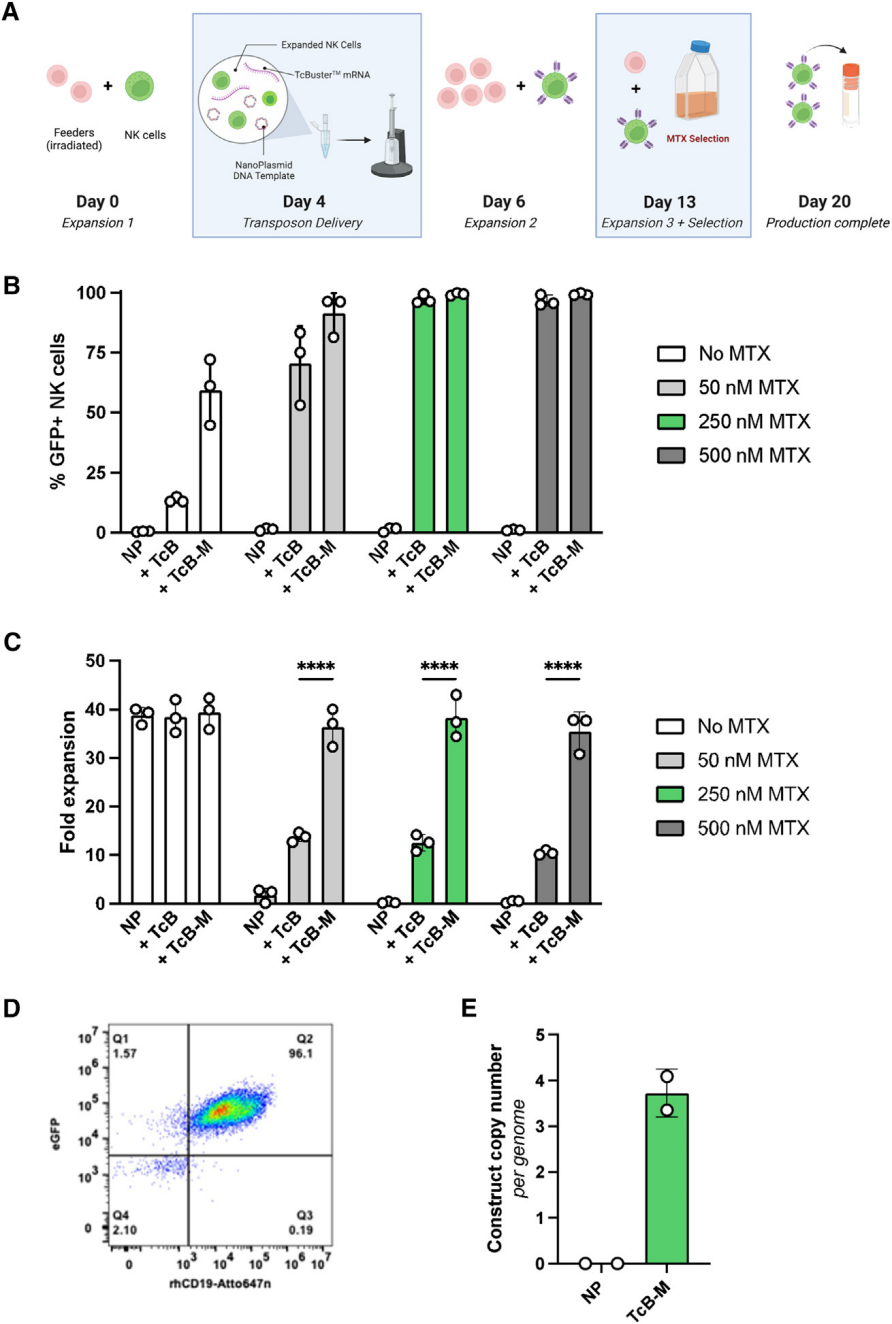
CD19-CAR-expressing NK cells are optimally generated with TcB-M and can be expanded to clinically relevant doses within 20 days CAR-positive cells were engineered with TcB-M and selected with 250 nM MTX (A–E). (A) Diagram of engineering and selection pipeline. (B) Construct expression post engineering and expansion with dose-escalation of MTX. (C) Fold expansion of engineered CAR-NK cells based on input cell number to final production numbers. (D) Flow analysis showing recombinant hCD19-Atto protein staining and co-GFP expression in engineered CAR-NK cells. (E) Results from construct insertion copy-number analysis as determined by ddPCR (SD shown, ∗∗∗∗*p* < 0.0001).

Selection of engineered immune cells for the purpose of therapy can be desirable, especially if there is a need to measure the impact of a dose-response from a pure population or if there is a requirement to only deliver engineered cells. One such way to provide selective pressure is to include an antifolate such as methotrexate (MTX), which inhibits wild-type dihydrofolate reductase (DHFR) that is essential for cell growth and proliferation.^40^^,^^41^ This approach is directly translatable as it is already being used clinically to enrich CAR-expressing T cells (NCT04483778). Our transposon cargo contained an MTX-resistant DHFR mutant (L22F, F31S),^42^ allowing us to select and expand NK cells that had undergone stable transposon integration. This selection step was thus incorporated into our production process during an additional round of feeder cell-mediated expansion (Figure 4A). Specifically, three doses of MTX were evaluated to determine the minimal dose required to kill integration negative cells and enrich engineered cells while maintaining high cell viability and recovery (Figures 4B–4D). An optimal dose of 250 nM MTX completely killed control cells (Figure 4C) and enriched cells engineered with either TcB or TcB-M to >99% GFP+ (Figure 4B). CD19-CAR expression was confirmed by staining cells with Atto 647N-labeled recombinant human CD19 (Figure 4D). This optimized protocol achieved manufacturing in ∼20 days and resulted in 99.2% (±0.5%) CAR+ NK cells expanded 1,380-fold (±104.4) from electroporation input (Figure S3). Copy-number analysis through ddPCR showed that an average of 3–4 CAR constructs were integrated into the genome of cells engineered with TcB-M (Figure 4E).

### Efficient engineering of CAR-T cells using hyperactive *TcBuster* and selection with methotrexate

**Figure 5:**
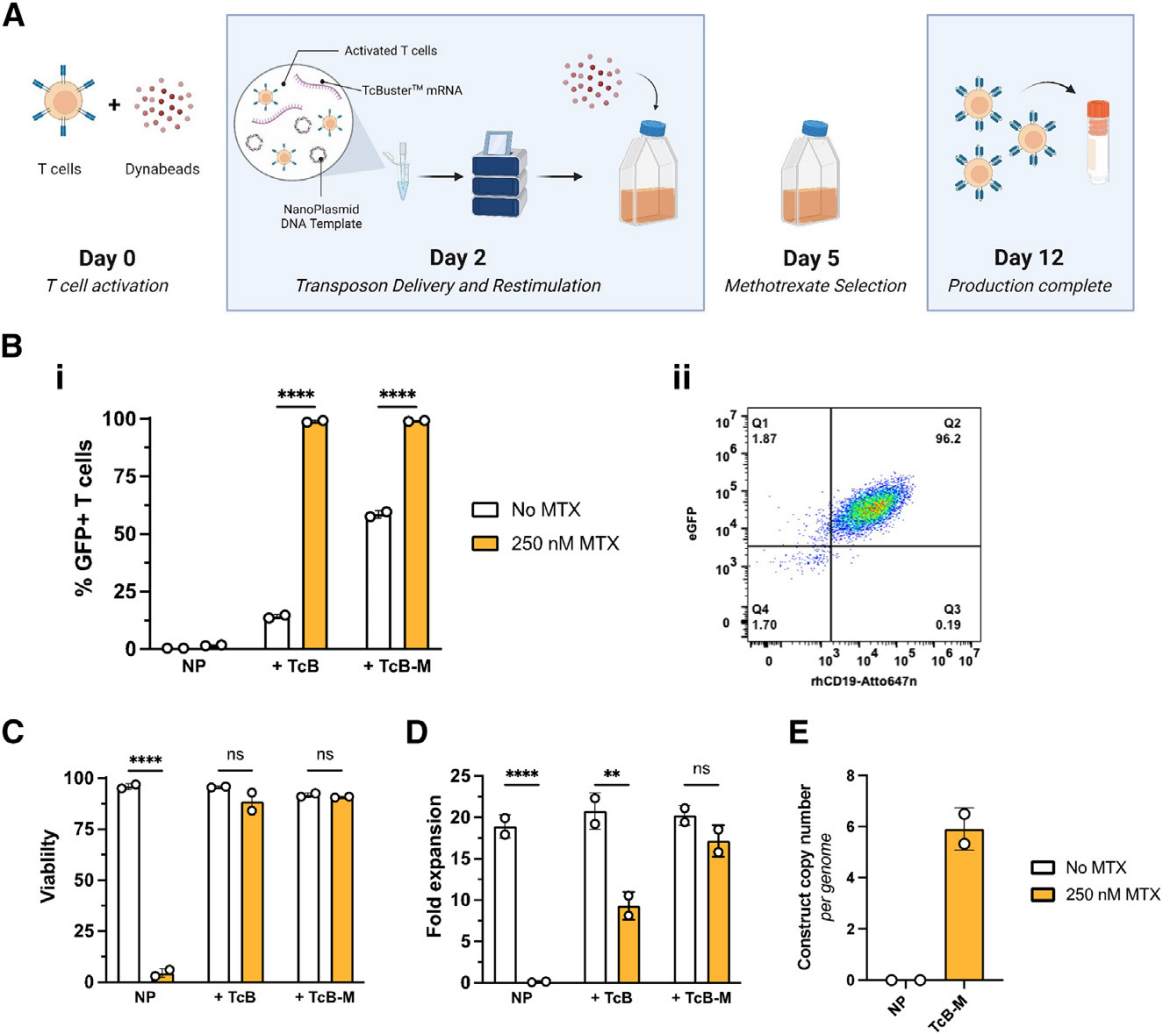
CD19-CAR-T cells can be generated using *TcBuster* transposition and expanded to therapeutic dosage levels (A) Engineering pipeline for the production of CAR-T cells using *TcBuster*: Primary human peripheral blood T cells are activated with ⍺CD3/⍺CD28 DynaBeads for 2 days. On day 2, DynaBeads are removed, and T cells are electroporated with transposition reagents and subsequently returned to DynaBead-containing cultures. Three days after electroporation, MTX is added to the media at a concentration of 250 nM. Seven days after MTX selection, production is complete and T cells are cryopreserved. (B–D) Production was performed with and without MTX selection. At the end of production, T cells were examined for construct expression through GFP expression (i) and recombinant CD19 protein staining (ii) measured by flow cytometry (B), cell viability was measured by trypan blue exclusion (C), and counts and fold expansion from electroporation input was calculated (D). (E) Results from construct insertion copy-number analysis as determined by ddPCR (SD shown, ∗∗∗∗*p* < 0.0001).

As NK and T cells have alternative and complementary roles in immune surveillance (reviewed in Meza Guzman et al.^43^ and Rao et al.^44^), it is possible that other groups may want an adaptive immune cell chassis for cellular therapy development and testing. We stimulated CD3+ primary human T cells from multiple donors with αCD3/αCD28 DynaBeads for 2 days and electroporated them with the transposon plasmid alone or in combination with TcB or TcB-M mRNA. Three days post-electroporation, we selected for transposon integration with or without 250 nM MTX for an additional 7 days, for a total production timeline of 12 days (Figure 5A). As with NK cells, we observed high-level integration without MTX (14% for TcB and ∼59% for TcB-M) and successful enrichment (>99% CAR+) of engineered T cells with MTX while maintaining high cell viability and co-expression of GFP and CD19-CAR (Figures 5Bi and 5Bii, 5C, and 5D). Importantly, we found that, post-manufacturing, between five and six copies were integrated into CAR-T cells engineered with TcB-M (Figure 5E). Overall, these data show that T cells can be rapidly and robustly manufactured with large, functional cargo utilizing hyperactive TcB-M in a GMP-compliant electroporation platform.

### Site-integration sequencing analysis

We conducted molecular analysis of TcBuster genomic insertion sites in both CAR-NK and CAR-T engineered cells using custom probe-based sequence capture (Table S1) and compared them to existing integration-site datasets for lentivirus and SB and PB transposon systems.^23^^,^^45^^,^^46^ Overall, the median distance to a transcriptional-start site for TcB-M integrations (18.023 kB ± 2.033 kB) is greater than reported for lentiviral integration (14.0 ± 0.3 and 11.2 ± 2.2), but not as distant as either PB or SB systems (23.6 ± 6.1 and 33.8 ± 5.1, respectively) (Table S2). Overall, this insertion site analysis reveals that TcB-M inserts transposons more randomly than lentiviral methods, and is similar to other transposase systems like SB and PB. Overall TcB-M integrations are more than twice as likely to occur outside of a transcript, and less than half as likely to integrate into a coding exon compared with published reports of lentiviral integration. While lentiviral methods show a strong bias to integrate into transcripts, transposase-based integration, including that of TcB-M, has minimal bias and trends closer to a random integration profile. (Table S2).

### Functional validation of CAR-NK and CAR-T cells

**Figure 6:**
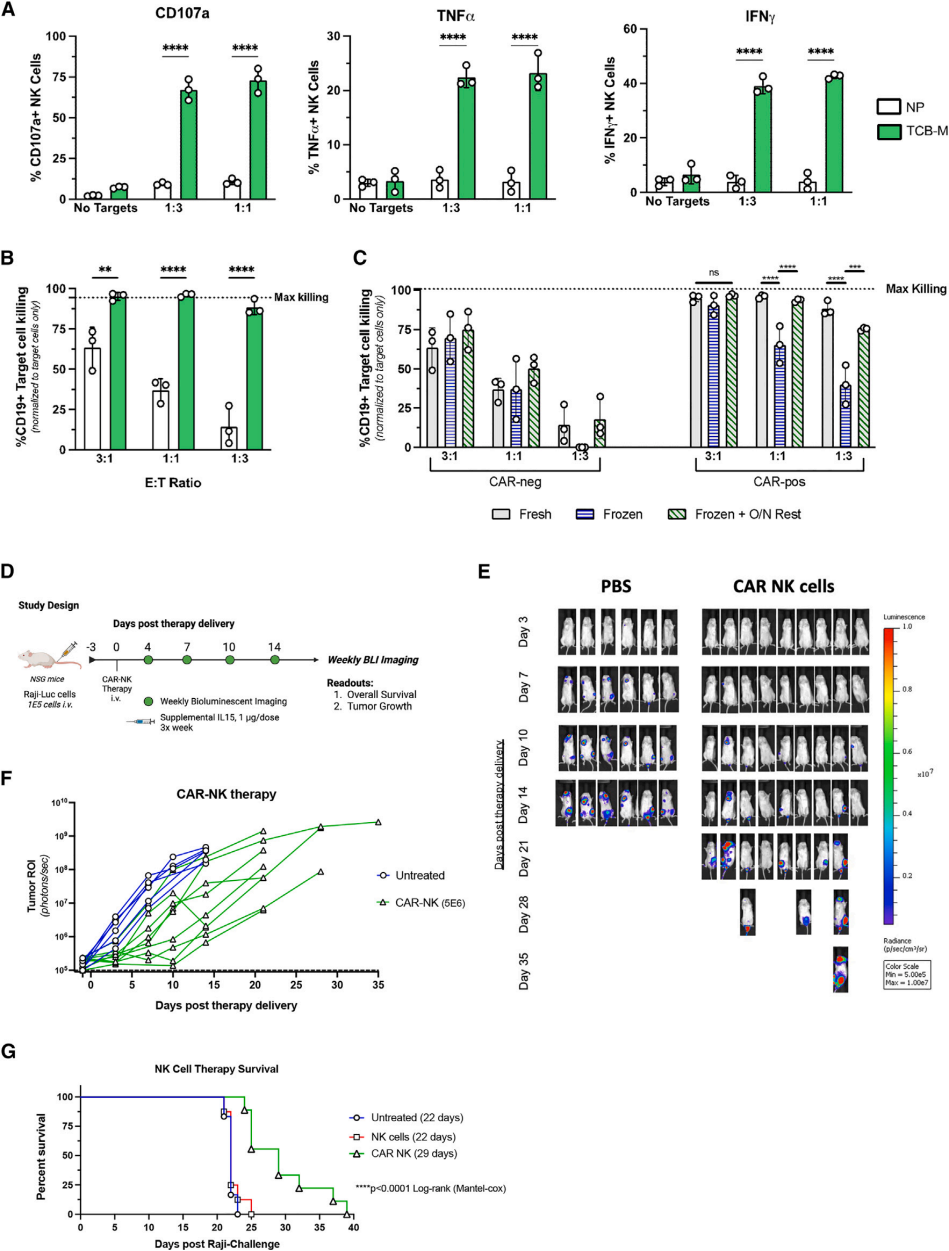
CAR-NK *in vitro* functionality and can improve survival in a preclinical model of Burkitt lymphoma with a single dose CAR-positive cells were engineered with TcB-M and selected with 250 nM MTX. CAR-negative control cells were electroporated with NP alone and were not selected with MTX. CAR-positive and CAR-negative NK cells were co-cultured with luciferase-expressing CD19+ Raji cells at the indicated E:T ratios for 5 h (A) or for 24 h (B, C). Raji-bearing mice were treated with PBS (*n* = 6), NK cells (*n* = 8, two donors) or CAR-NK cells (*n* = 9, two donors) at day 3 post tumor injection and monitored for tumor growth through bioluminescence imaging and overall survival (D–G). (A) CAR-NK cells show significant upregulation of degranulation markers and effector cytokines when co-cultured with CD19-expressing Raji cells. (B) Target killing of Raji-luc cells is significantly enhanced in CAR-expressing NK cells at 24 h. (C) Killing assay results for freshly engineered CAR-NK, CAR-NK thawed cells from cryopreserved state, or CAR-NK thawed cells that were rested overnight in cytokine-containing media prior to the start of assay. (D) Visualization of *in vivo* experimental design. (E) Bioluminescent images of mice over the course of the study at indicated days post therapy delivery from PBS and CAR-NK groups. (F) Individual tumor growth of mice receiving PBS (*n* = 6) and CAR-NK cells (*n* = 9, two donors). (G) Kaplan-Meier survival curve of Raji-tumor-bearing mice receiving single-dose monotherapy of CAR-NK cells, donor-matched non-CAR NK cells, or PBS. *In vitro* statistical analyses were done per culture condition by comparison to the non-CAR engineered cells using a one-way ANOVA followed by Dunnett’s multiple comparisons test (*n* = 3 independent biological donors) (SD shown, ns = not significant, ∗∗*p* < 0.01, ∗∗∗*p* < 0.001, ∗∗∗∗*p* < 0.0001). Survival significance was determined by a Log rank (Mantel-Cox) test.

We next engineered both NK and T cells with TcB-M and selected to >99% CAR+ with MTX for testing in functional assays against the CD19-expressing Raji Burkitt lymphoma cell line. NK or T cells electroporated with the transposon plasmid alone (lacking transposase mRNA) and expanded without MTX selection served as controls. Of note, no residual copies of transpon were detected via ddPCR in these cells and they did not express detectable CAR (Figures 4E and 5E). First, CAR-negative or CAR-positive NK cells from three separate donors were co-cultured with Raji target cells for 5 h at various effector-to-target (E:T) ratios and analyzed for levels of functional degranulation and effector cytokine production through intracellular cytokine staining. CAR-NK cells showed significantly more CD107a on their cell surface and produced more tumor necrosis factor (TNF)-α and interferon (IFN)-γ than CAR-negative NK cells after coculture (Figure 6A). In luciferase-based killing assays, CAR-NK cells eliminated over 90% of Raji cells in 24 h at the lowest E:T ratio of 1:3 (Figure 6B). These data show robust functionality of CAR-NK cells against CD19-expressing target cells post engineering and selection.

With an ultimate goal of deploying our transposon manufacturing pipeline for clinical use, there is a requirement for cryopreservation and banking of manufactured CAR-NK cells. Recent studies have shown that while cryopreservation may have little effect on NK viability, it can cause a loss of cytotoxic function.^47^ Thus, we tested cytotoxicity of CAR-NK cells cryopreserved in CryoStor (R) CS10 (StemCell) immediately after thaw or after overnight culture in media containing 100 IU/mL IL-2. Although cytotoxicity of CAR-NK cells immediately after thaw was reduced compared with fresh cells, target cell killing was still efficient at higher E:T ratios (3:1) (Figure 6C). Cytotoxicity of frozen CAR-NK cells was restored to that of fresh cells after an overnight (16-h) rest in media containing IL-2. We did not observe a significant reduction in cell number or viability during the rest period (Figures S5A and S5B). Furthermore, in-depth phenotyping of three independent donor CAR-NK showed a significant increase in NKp46 and NKp44 in overnight IL-2 rested cells compared to freshly thawed ones, suggesting rest increases two surface receptors directly correlated with NK cell cytotoxicity^48^^,^^49^^,^^50^^,^^51^ (Figure S6).

To examine the efficacy of our engineered CAR-NK cells *in vivo*, we tested single-dose CAR-NK in a xenograft model of Burkitt lymphoma (Figure 6D). Tumor-bearing animals were given either 5E6 CAR-NK cells (*n* = 9) or non-CAR-expressing control NK cells (*n* = 8) from two donors and tumor growth was monitored using bioluminescence imaging. Mice receiving CAR-NK and NK cells also received supplemental IL-15 at 1 μg/dose intraperitoneally three times weekly to promote *in vivo* persistence of NK cells. Mice that received CAR-NK therapy showed some level of tumor control over NK only and PBS mice, leading to significantly improved survival of CAR-NK (29 days) over PBS (22 days) groups or matched non-CAR-therapy cells (22 days for NK only) (Figures 6E–6G) (Figure S7). Of note, even though the single dose of 5E6 CAR-NK treatment did not result in full tumor control or clearance, analysis of cells in the bone marrow and spleen revealed the cells were still present and nearly 100% CAR-cassette expressing but showed high levels of T cell immunoreceptor with Ig and ITIM domains (TIGIT) expression (Figure S8), suggesting the CAR-NKs were able to home to the bone marrow niche and persist with exogenous IL-15 supplementation but may have hit exhaustion.

**Figure 7:**
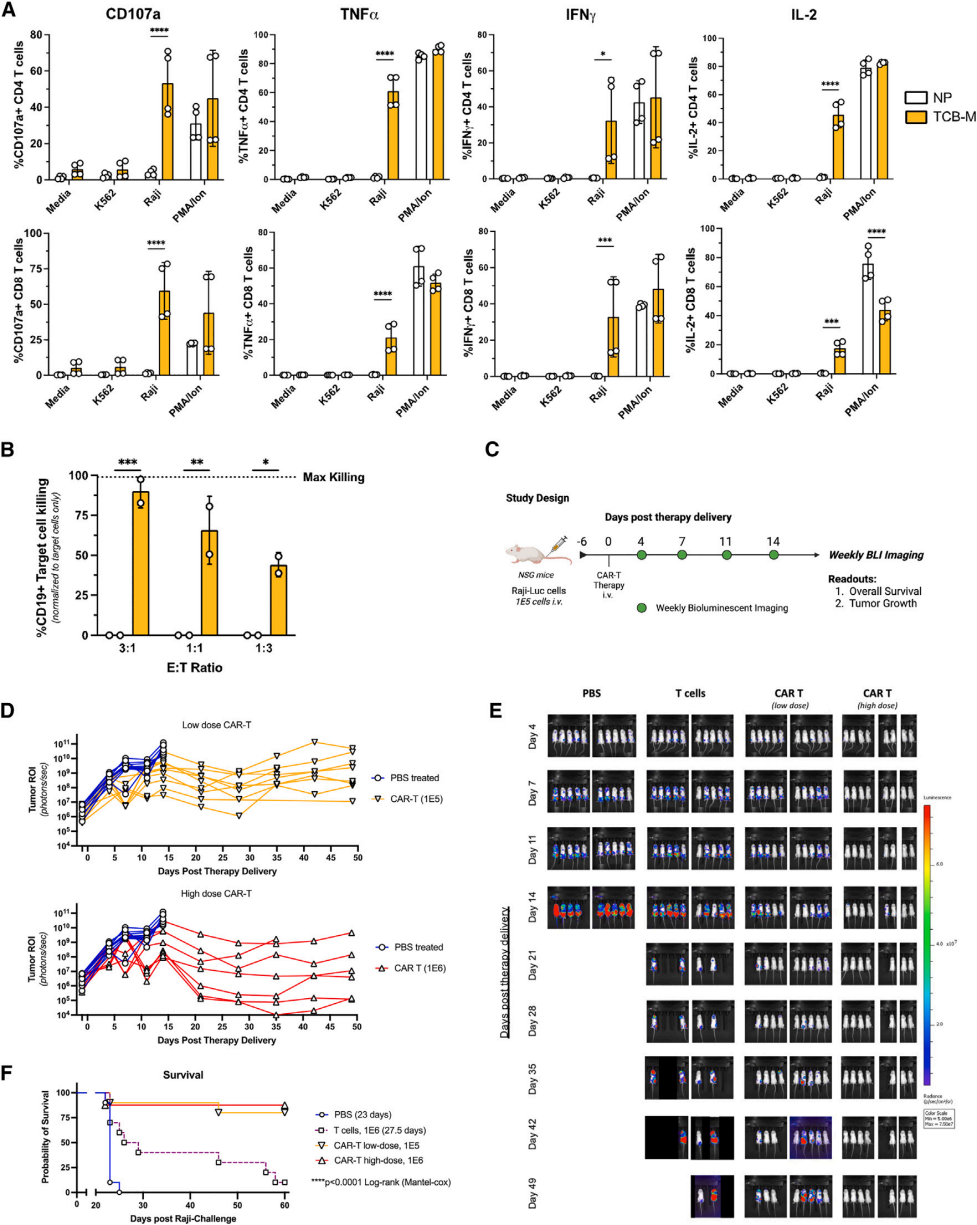
CAR-T cells show robust *in vitro* functionality and can significantly improve survival in a preclinical model of Burkitt lymphoma with a single dose CAR-positive cells were engineered with TcB-M and selected with 250 nM MTX. CAR-negative control cells were electroporated with NP alone and were not selected with MTX. CAR-positive and CAR-negative T cells were co-cultured with luciferase-expressing CD19+ Raji cells at 1:1 (E:T) for 8 h (A) or 24 h at indicated E:T (B) to assess functional cytokine production and cytotoxic activity. Raji-bearing mice were treated with PBS (*n* = 10), T cells (*n* = 10, two donors), low-dose CAR-T cells (*n* = 10, two donors), or high-dose CAR-T (*n* = 8, two donors) at day 6 post tumor injection and monitored for tumor growth through bioluminescence imaging and overall survival (C–F). (A) CD4+ (Top) and CD8+ (Bottom) CAR-T cells show significant upregulation of degranulation markers and effector cytokines when co-cultured with CD19-expressing Raji cells, a phorbol myristate acetate (PMA)+ionomycin-positive stimulus control, and not CD19-negative K562 cells. (B) Luc-based killing assay of 24-h coculture of CAR-T with Raji-luc cells. (C) Visualization of *in vivo* experimental design. (D) Individual tumor growth from Raji-bearing mice treated with PBS (*n* = 10), low-dose CAR-T (1E5 cells, *n* = 10, two donors), or high-dose CAR-T (1E6, *n* = 8, two donors). (E) Bioluminescent images of mice over the course of the study at indicated days post therapy delivery from all groups. (F) Kaplan-Meier survival curve of Raji-tumor-bearing mice receiving single-dose monotherapy of CAR-expressing T cells, donor-matched non-CAR-T cells, or PBS. *In vitro* statistical analyses were done per indicated culture condition by comparing CAR-T engineered cells and matched donor T cells lacking CARs using a one-way ANOVA followed by Dunnett’s multiple comparisons test (*n* = 2 independent biological donors) (SD shown, ns = not significant, ∗*p* < 0.05, ∗∗*p* < 0.01, ∗∗∗*p* < 0.001, ∗∗∗∗*p* < 0.0001). Survival significance was determined by a Log rank (Mantel-Cox) test.

As with our CAR-NK cells, transposon-engineered and MTX-selected CAR-T cells from multiple donors showed strong functional responses against CD19(+) Raji cells, but not against control CD19(−) K562 cells. Specifically, CD107a+, TNF-α, IFN-γ, and IL-2 expression were markedly increased after coculture with Raji cells in both CD4+ (Figure 7A top) and CD8+ T cell populations (Figure 7A bottom). Engineered CAR-T exhibited robust cytotoxicity against Raji cells after 24 h at all E:T ratios (Figure 7B). In contrast to CAR-NK cells, cytotoxicity was not significantly different between freshly engineered, frozen, or thawed and rested CAR-T to eliminate targets (Figure S9). Next, we evaluated the *in vivo* function of TcB-engineered CD19 CAR-T cells in NSG mice engrafted with Raji. Both a low dose (1E5 CAR-T, *n* = 10 from two donors), and higher dose (1E6 CAR-T, *n* = 8 from two donors) of CAR-T cells were able to significantly delay tumor growth and improve survival (Figures 7D–7F) (Figure S10). Together, these data demonstrate that hyperactive *TcBuster* (TcB-M) can generate both CAR-NK and CAR-T cells capable of robust functional responses against CD19+ tumor targets both *in vitro* and *in vivo.*

## Discussion

Current methods for clinical manufacture of cellular therapies, including CAR-NK and CAR-T cells, largely rely on viral vectors for CAR integration. There are significant drawbacks associated with viral vectors for clinical manufacture of cell therapies, including (1) costly, complex, and protracted vector production processes that are largely intractable in the academic setting; (2) limited capacity to deliver large, complex genetic cargo; and (3) documented risk of insertional mutagenesis that recently prompted the Food and Drug Administration (FDA) to issue a black box warning for several commercial CAR-T products engineered with viral vectors.^52^ Transposon systems are an attractive alternative to viral vectors due to the rapid and cost-effective production of clinical transposon reagents and a preferable integration profile with reduced preference for integration near gene regulatory elements.^23^

The goal of the present work was to develop a hyperactive form of the *TcBuster* transposon system and establish proof-of-principle for its ability to engineer primary human cells for the purpose of cellular therapies. *TcBuster* is part of the hAT family of transposases, which is one of the most ubiquitous families of transposases found in nature.^25^ Successful transposon elements proliferate and diversify by propagating vertically within a single host species lineage and by horizontal transfer between species.^53^ To avoid genomic instability and excessive toxicity to their host, transposases evolved with limited activity and mechanisms for self-regulation, which is likely the rationale for most transposon systems having low activity in human cells.^54^^,^^55^ As stated before, previous work on the *SB* and *PB* transposon systems have demonstrated the feasibility to enhance the efficiency of DNA transposon systems via incorporation of phylogenetically conserved amino acids, structure-guided mutagenesis, and gene-shuffling approaches.^31^^,^^32^ Therefore, we reasoned that we could deploy these methods to construct a new, hyperactive *TcBuster* system.

To this end, we performed amino acid substitutions based on conserved sequences of hAT family transposases and carried out a high-throughput gene-shuffling screen of the native *TcBuster* transposase*.* Primary screening showed little improvement with single and some combinatorial substitutions; however, by shuffling the 108 identified amino acid substitutions via DNAse treatment and primerless assembly PCR, we were able to identify additive and synergistic combinations, culminating in the identification of our hyperactive transposase mutant (TcB-M) with 3- to 4-fold (∼20% to >60%) enhanced transposition activity. Interestingly, this variant enzyme contained a few substitutions, which although were not very enriched in the high-throughput screen, were identified as being hyperactive in initial single substitution screens, demonstrating that the context of the mutation likely plays a significant role in its function. Previous approaches in screening included labor-intensive methods of testing 2,000 variants individually as was done for *SB100X* in HeLa cells.^31^ For the hyperactive form of *PB*, a bulk population-based high-throughput screening in yeast was done to examine a library of ∼10,000 variants.^32^ However, a majority of the hyperactive variants obtained from the yeast screen were not hyperactive in mammalian cells. A follow-up study that used a similar yeast screening for optimized hyPBase found 15 candidates that had higher transpose activity compared with optimized hyPBase (bz-hyPBase) in yeast, which then yielded four mutants showing higher transposase activity in mammalian cells among 3,000 hyPBase mutants screened.^56^ Here, our new approach allowed us to examine a library of 3 million unique TcBuster variants directly in mammalian cells, which is considerably more diverse than previous transposase engineering efforts.

We then used both the original TcB and TcB-M transposases to deliver an MND-driven expression cassette containing a CD19-CAR, DHFR mutein, and eGFP to primary human PBL NK and T cells and provide our optimized protocols to do so. To date, the use of transposons in NK cells has potentially been limited by DNA toxicity due to a type I IFN response.^2^^,^^57^^,^^58^ To avoid this, we delivered the transposase as modified mRNA^59^ and the transposon via nanoplasmid vectors, which have a small backbone, high supercoiling, and are readily scaled under cGMP compliance.^60^ While we did not directly examine the levels of cytosolic DNA-sensing proteins or their signaling in NK cells during prolonged activation here, we did recently publish a study where we identified a temporal window/goldilocks zone that exists during T cell activation that allows them to be more amenable to receiving small backbone DNA cargos for the purposes of cellular engineering.^38^ We also optimized the activation, electroporation, recovery, and expansion conditions to achieve nearly 50% integration efficiency in primary NK cells without selection. Interestingly, one of the greatest improvements made in our base-level engineering was including pre-treatment of NK cells with a broad-spectrum RNase inhibitor targeting RNase A, RNase B, and RNase T2 prior to electroporation in our workflow.^39^ This alone enhanced transposition efficiency from 24.05% (±1.36%) to 49.63% (±4.64%). Degradation of the transposase mRNA by RNases is a phenomenon that has been noted by our group within other primary human immune cells.^61^ However, there are very limited data available on the expression of ribonucleases in NK cells in resting or activated states. While outside the scope of this study, future work that directly examines the changes of both DNA sensing and the RNase enzyme repertoire in primary NK cells throughout activation may reveal specific pathways or targets that can be manipulated for further enhancements to engineering.

Next, to allow flexibility of a cell-culture-based selection method during engineering, we also included an MTX-resistant DHFR mutein (L22F, F31S) in our cargo.^42^ This allowed us to enrich our engineered population using MTX, without the need for GMP-compliant cell sorting facilities or clinical-grade monoclonal antibodies. Furthermore, MTX is already being used in an *ex vivo* CAR-engineered process as part of an active clinical trial (NCT04483778). Overall, we developed a CAR-NK cell manufacturing protocol that includes 4 days of feeder cell activation before electroporation, re-expansion 2 days after electroporation, and a third expansion with MTX selection. This process results in ∼1,400-fold expansion from electroporation input when cells are engineered with TcB-M. Current clinical trials using CAR-NK cells have treated patients with doses ranging from 1E5 – 1E7 cells/kg of body weight.^62^ Thus, the average North American patient (80 kg) would receive a dose of 8E6 to 8E8 NK cells. From our protocol, a single dose of 1E7 cells/kg could be produced in 20 days from a starting number of just 5.8E5 NK cells. Given that apheresis products from healthy donors contain an average of ∼1E9 NK cells (10% of total PBMCs assuming a reported average of 13.6E9 cells),^63^ we could theoretically generate an average of 1,724 higher-end (1E7 cells/kg) doses from a single leukopak using our TcBuster transposon engineering pipeline. As CAR-T cells have been previously generated for preclinical and clinical use with a number of transposon systems, we also demonstrated the feasibility and high efficiency of their production using the TcB-M variant paired with selective outgrowth of engineered cells through MTX selection during expansion.^57^^,^^58^^,^^64^^,^^65^^,^^66^^,^^67^^,^^68^^,^^69^^,^^70^^,^^71^

Integration into active genes or near transcriptional-start sites is undesirable as it can disrupt cell function and, if the genes are oncogenes or tumor-suppressor genes, it could lead to malignancy. In fact this is no longer a hypothetical concern, as highlighted in a recent letter from the FDA.^52^ The FDA is currently investigating serious risk of T cell malignancy following commercial BCMA-directed or CD19-directed autologous CAR-T cell immunotherapies engineered with lentivirus. Reducing the bias of transgene insertion toward active genes and transcriptional-start sites reduces the risk of malignancy, suggesting TcB-M and other transposase-based gene delivery methods may provide a safer alternative to lentiviral gene delivery as we have shown through site-integration sequencing in this study and others.^72^

We next validated the function of both CAR-NK and CAR-T cells produced using our optimized protocols in standard *in vitro* and *in vivo* assays. CAR-NK cells showed high levels of degranulation, inflammatory cytokine production, and target cell killing compared with CAR-negative controls. Interestingly, it has been shown that cryopreservation may lead to reduced cytotoxicity of NK cells.^47^ An off-the-shelf product requires cryopreservation and banking of engineered cells. We did observe reduced cytotoxicity of CAR-NK cells immediately after thaw, but this phenotype was rescued after an overnight rest in media containing 100 IU/mL IL-2. Flow cytometric analysis of post-thaw and post overnight rest CAR-NK phenotyping showed increases in NKp46 and NKp44 within overnight IL-2-treated CAR-NK cells. NKp46 is directly correlated to increased cytotoxic function of NK cells, while NKp44 is considered an activation marker of NK cells and its expression is also correlated to cytotoxic function.^48^^,^^49^^,^^50^^,^^51^ However, it is likely not feasible to include this type of overnight rest period in a clinical protocol. We observed efficient target cell killing immediately after thaw when we used higher E:T ratios. Therefore, instead of implementing an overnight rest in a clinical protocol, it is likely more practical to simply deliver a higher dose of cells. This is feasible as the administration of CAR-NK cells clinically has not been associated with cytokine release syndrome, neurotoxicity, or graft-vs.-host disease, and the maximum tolerated dose has not been reached.^62^

As with CAR-NK cells, CAR-T cells also showed a functional profile of activation and functionality against target cells, highlighting that our manufacturing protocol produces high-quality CAR-T cells. We did note that there was no functional difference in cryopreserved vs. freshly manufactured cells in killing assays, suggesting that selection with MTX directly preceding cell cryopreservation does not negatively impact function post freeze-thaw. Previous studies from our lab and colleagues utilizing non-optimized TC Buster engineering pipelines showed the ability of TcB-M to generate functional BAFF-L CAR-T cells; however, there have yet to be studies showing *in vivo* efficacy of CAR-NK cells derived from TcB-M engineered primary NK cells.^72^^,^^73^ Here, we found that the utilization of either CAR-NK or CAR-T in a single-dose monotherapy for Raji-bearing NSG mice showed significant improvement in overall survival. While CAR-T showed tumor reduction and control at 1E6 cells per dose through the duration of the study, CAR-NK cells from two donors showed little control of *in vivo* tumor growth. Both analysis of cells in the bone marrow and spleen revealed NK cells were still present and nearly 100% CAR-cassette expressing, suggesting the CAR-NKs were able to home to the bone marrow niche and persist through the study. Interestingly, CAR-NKs showed near-100% expression of TIGIT, which in NK cells has been directly shown to reduce cytotoxicity during chronic tumor stimulation unless countered with an anti-TIGIT antibody blockade.^74^ This failure to control at single doses seems to be a common occurrence with CAR-NK *in vivo* studies as effective tumor control is either seen when tumor and therapy are delivered simultaneously or multiple, high doses of (5-10E6) of CAR-NK are delivered through the course of the study.^75^^,^^76^^,^^77^^,^^78^ Recently, Basar et al. found that this failure is in part linked to trogocytosis of CAR antigens from target cells by subsets of CAR-NK cells, which leads to fratricide and poor therapy outcomes.^79^ Given the flexibility of cargo sizes with transposon-based engineering, it is likely possible to include IL-15 cytokine armoring and/or the inclusion of a second inhibitory CAR in the same transposon vector to prevent trogocytosis-mediated fratricide and improve outcomes.^79^

It is of note that our detailed pipelines for TcB-M engineering of CAR-NK and CAR-T cells is not limited to the delivery of CAR cassettes; other modifications have been proposed to enhance aspects of immune cell activity including persistence, migration, and cytotoxicity. These include, but are not limited to, the introduction of self-stimulating cytokine receptors,^80^ strong activating receptors, dominant negative versions of effector cell inhibitory receptors,^81^ or regulatory CARs that prevent self-targeting due to trogocytosis of target cell antigens.^82^ Such modifications could be used in combination with CAR delivery to create a cellular immunotherapy expertly equipped to kill a broad range of tumor types. Furthermore, this non-viral system could be rapidly deployed to test different constructs and cell types for the treatment of diseases, insertion of reporter plasmids in cell lines of interest, and even as a tool for screening cancer mutations or genes that impact normal cellular functions. As a testament to the speed that this tool ushers therapies into the clinic, as of this publication at least three therapies manufactured with TcB-M have received FDA investigational new drug approval and are in the clinic for phase 1 testing (NCT05312801, NCT05546723, and a third has been approved for treating lupus in phase 1 as of December 2023). Overall, our work provides a robust method to generate and screen for hyperactive transposase moieties as well as a detailed engineering pipeline for robust delivery of multicistronic, large cargo via transposition in primary human PBL NK and T cells using the neon system and Amaxa 4D, respectively. Our proof-of-principle results demonstrate that CAR-expressing NK and T cells can be enriched using MTX selection at clinically relevant doses *ex vivo*, while maintaining high viability/function, and large-scale manufacturing can be accomplished in a matter of weeks. This approach represents a versatile, safe, and cost-effective option for the manufacture of CAR-NK, CAR-T, and other future cellular therapies using this novel hyperactive *TcBuster* system.

## Materials and methods

### General reagents

The following antibodies, proteins, and dyes were used: APC- or PE-conjugated anti-CD56 (clone REA196, Miltenyi Biotec or clone NCAM, BioLegend), PE-conjugated anti-CD3 (clone SK7; BD Biosciences), BV605-conjugated anti-CD4 (clone RPA-T4; BioLegend), PE-conjugated anti-PD-1 (clone PD1.3; Miltenyi), BV650-conjugated anti-CD8 (clone RPA-T8; BioLegend), BV421-conjugated anti-human CD45 (clone 2D1; BioLegend), BV605-conjugated anti-mouse CD45 (clone 30-F11; BioLegend), Atto 647N-conjugated recombinant human CD19 (Bio-Techne), Brilliant violet 421-conjugated anti-IFN-γ (clone 4S.B3; BioLegend), APC-labeled anti-TNF-ɑ (clone Mab11; BioLegend), anti-CD107a (clone H4A3; BD Biosciences), PE-conjugated anti-NKG2A (clone S19004C; BioLegend), PE/Cy7-conjugated anti-NKp30 (clone P30-15; BioLegend), APC-conjugated anti-NKG2D (clone 1D11; BioLegend), AF700-conjugated anti-NKp46(clone 9E2; BioLegend), BV510-conjugated anti-CD161 (clone HP-3G10; BioLegend), PE-conjugated anti-CD96 (clone NK92.39; BioLegend), PE/Cy7-conjugated anti-Tim3 (clone F38-2E2; BioLegend), APC-conjugated anti-CD94 (clone DX22; BioLegend), AF700-conjugated anti-KIR3DL1.1 (clone DX9; BioLegend), BV510-conjugated anti-OX-40 (clone Ber-ACT35; BioLegend), PE-conjugated anti-NKp44 (clone P44-8; BioLegend), PE/Cy7-conjugated anti-2B4 (cloneC1.7; BioLegend), APC-conjugated anti-CD158 (clone HP-MA4; BioLegend), AF700-conjugated anti-CD16 (clone 3G8; BioLegend), BV510-conjugated anti-CD2 (clone PRA-2.10; BioLegend), PE-conjugated anti-NKG2C (clone S19005E; BioLegend), PE/Cy7-conjugated anti-TIGIT (clone A15153G; BioLegend), AF700-conjugated anti-CD38 (clone HIT2; BioLegend), BV510-conjugated anti-LAG-3 (clone 11C3C65; BioLegend), SYTOX Blue dead cell stain (ThermoFisher), and Fixable viability dye eFluor 780 (eBioscience). Flow cytometry assays were performed on a CytoFLEX S flow cytometer (Beckman Coulter) or a BD Bioscience LSR Fortessa and all data were analyzed with FlowJo version 10.4 software (FlowJo LLC).

### Biological resources

*Cell lines:* HeLa, 293T, and Raji cells were acquired from ATCC and cultured in RPMI-1640 media supplemented with 10% FBS and Pen-Strep. NK feeder cells were generated in-house using previously published methods^83^

*Donor T and NK Cell Source and Isolation:* Peripheral blood mononuclear cells (PBMCs) from de-identified healthy human donors were obtained by automated leukapheresis (Memorial Blood Centers, Minneapolis, MN). CD3+ T cells or CD56+CD3− NK cells were isolated from the PBMC population using the EasySep Human T cell Isolation Kit or EasySep Human NK Cell Isolation Kit (STEMCELL Technologies, Cambridge, MA). T cells were frozen at 1-2E7 cells/mL and NK cells were frozen at 5E6 cells/mL in CryoStor CS10 (STEMCELL Technologies, Cambridge, MA) and thawed into culture as needed. Samples were obtained after informed consent with approval from the University of Minnesota Institutional Review Board (IRB 1602E84302).

### Plasmid construction

TcBuster transposase ORF, 5′ ITR, and 3′ ITR sequences were obtained from NCBI (Genebank Accession number DQ481197). The TcBuster transposase sequence was synthesized and codon optimized for mammalian cells at Genscript. The sequences flanking the TcBuster ORF are considered the TcBuster ITRs.^25^ These sequences were synthesized as reverse complements that flank a multiple cloning site, allowing cassettes CMV, (mCherry/hygromycin/or GFP) with BGH polyA to be subsequently cloned in. For splice acceptor transposons, CMV was replaced with a splice acceptor. Mammalian codon optimized TcBuster was cloned into pcDNA 3.1. SB100X and HyPBase sequences were synthesized and codon optimized for mammalian cells by Genscript.

### Lentivirus library generation

The TcBuster variant library was moved into a lentiviral transfer vector with an IRES-Puromycin to titer functional virus. Lentivirus was produced by lipofectamine 3000 (Invitrogen) transient transfection of 293T cells with the TcBuster Lentiviral transfer vector, PMD2.g, and psPAX2. Twenty-four hours following transfection, media was changed and cells were moved to 30°C to slow proliferation. Seventy-two hours following transfection, media was collected, and virus was concentrated with Lenti-X Concentrator (Clontech, 631231). Functional viral titer was determined by counting the number of puromycin-resistant colonies and calculated by TU/mL = number of colonies/total volume in the well (mL) × dilution factor. Upon determining functional titer, 20 million HeLa cells were transduced at an MOI of 0.3 with the TcBuster Lentiviral Library. Forty-eight hours following transduction, cells were treated with 1 μg/mL of puromycin. Cells were expanded and maintained at high numbers to preserve library diversity.

### High-throughput TcBuster activity screen

Twenty million HeLa cells were transfected with either 50 ng of hygromycin transposon and splice acceptor driven, GFP, and mCherry transposons each or 500 ng of each. Twenty-four hours following transient transfection, HeLa cells were treated with 400 μg/mL of hygromycin to kill any transposition negative cells. Five days following transfection, HeLa cells were FACS sorted with a BD FACS ARIA II.

### Testing TcBuster variants

A total of 250,000 HeLa cells were seeded and 24 h later transiently co-transfected with a 1 μg TcBuster variant and 0.5 μg GFP transposon. Ten days following transfection, cells were measured for stable GFP expression via flow cytometry on a BD Bioscience LSR Fortessa as a measure of transposition efficiency (*n* = 3).

### TcBuster variant library creation

Plasmids containing individual point mutations were obtained from Creative Biostructure from a failed attempt to shuffle the mutations. Plasmids were digested using restriction enzymes to select the small portion of the gene (200–600 base pairs [bp]) containing the mutation. We gel extracted each fragment and quantified them by nanodrop. We pooled 90 fmol of each fragment to be included and concentrated them by Zymo clean and concentrate 5 kit. DNAseI treatment: ∼500 ng of DNA mutant fragment pool was mixed in a 12.5-μL DNAseI digestion. The final buffer for DNAseI reaction contained 20 mM MnCl2 (note, MgCl2 results in DNAseI repeated single-strand nicking and more digestion, while MnCl2 results in double-strand breaks that limit the DNAseI cutting to ∼50-bp fragments), 50 mM TrisHCl (pH 7.4), and 0.5 mM CaCl2 (note, calcium is essential for DNAseI function, but is omitted from many previously published protocols). Digestion was carried out with 0.1 U DNAseI (NEB M0303S) for 90 min at 25°C and halted by incubating 10 min at 98°C. The resulting digestion was verified by gel electrophoresis and cleaned up using Monarch PCR & DNA Cleanup Kit using the oligonucleotide purification protocol variation. We next carried out assembly PCR using 40% of the digested gene pool, and 2X Q5 polymerase in a 20-μL reaction. Cycling parameters were initial denaturing step 98°C for 30 s, followed by 41 cycles of 98°C for 10 s/55°C for 20 s/72°C for 20 s + 1 s per cycle, with a final extension step of 72°C for 5 min. The assembled reaction was then used in a primed amplification reaction without any further purification. The 50-μL amplification reaction included 5 μL of the assembly reaction, 40 nM forward and reverse primers (o2541 and o2542), containing homology arms for assembly into the lenti vector via Gibson Assembly, and a low amount (2 nM) of spiked in linking primer (o2510) to allow amplification of any assembled genes without an intact 3′ end, and 2X Q5 polymerase master mix. Reaction parameters were as follows: initial denaturing: 98°C 30s, 30 cycles of 98°C 10s/69 °C C 10s/72 °C C 90s, followed by a final extension step of 72°C for 2 min.

We gel extracted the 2-kb band for the full-sized gene. The gene library was then inserted into the linearized lenti vector via Gibson Assembly, 200 ng of gel extracted gene library was mixed with 75 ng vector using an in-house *in vitro* recombination mix in a 20-μL reaction. The reaction was incubated at 50°C for 10 min, then returned to ice. Ten microliters of the Gibson Assembly was dialyzed on a 0.05-μM MCE membrane (MF-Millipore) in deionized water for 30 min. This was added to 100 μL Endura ElectroCompetent Cells (Lucigen) on ice. The mix was split into four 25-μL electroporations. Electroporation was done in a 1-mm cuvette, 25 μF, 200 Ω, 1.6 kV. Each electroporation was recovered in 2 mL of Lucigen recovery media, shaken at 37°C. The four reactions were pooled, pelletted, and plated. Dilution plates were made for estimating counts, and the remainder was plated across two 15-cm salt-free LB + carbenicillin plates. These were grown overnight at 30°C. The total transformants were 4.1 million colonies. To collect cells, 2 mL of salt-free LB media was added to the two undiluted plates, and they were combined. The cells were pelleted for 10 min at 3,000 × *g* and maxiprepped using NucleoBond Xtra Maxi EF kit per the manufacturer’s instructions, resuspending in 200 μL of water at a final concentration of 615 ng/μL.

### High-throughput mammalian library screen

Total counts of sorted cells can be found in Table S3.

### NGS library prep and analysis

Genomic DNA was extracted from preselected and sorted cells using QIAamp DNA Micro Kit (QIAGEN) and eluted in 0.1X TE buffer. DNA was amplified for sequencing by sequential nested PCR (Table S4). First, 50-μL PCR reactions were done with 1 μL extracted genomic DNA, 200 nM forward primer (o895), 200 nM reverse primer (o271), with 25 μL 2X Q5 polymerase master mix. Program parameters were as follows: initial denaturing at 98°C for 60 s, 35 cycles of 98°C 10 s/64°C 10 s/72°C 90 s, followed by final extension at 72°C for 2 min. This was cleaned up using Monarch PCR & DNA Cleanup Kit, and eluted in 20 μL water. A secondary PCR to gain enough material for sequencing was carried out at 100-μL scale. This PCR contained 50 ng DNA from the initial PCR, 100 nM forward primer (o2602), 100 nM reverse primer (o2603), and 50 μL Q5 polymerase 2X master mix. PCR parameters were initial denaturing at 98°C for 2 min, followed by 25 cycles of 98°C 10 s/72°C 90 s, followed by a final extension at 72°C for 2 min. These reactions were cleaned up using Monarch PCR & DNA Cleanup Kit, eluted in 40 μL of 10-mM TrisHCl, pH 8.0, and sent to Genewiz for PacBio CCS sequencing.

Reads were analyzed for presence of mutations using a custom Python script. Briefly, sequencing reads were filtered to only include full-length gene sequences that align to the reference wild-type sequence. The aligned sequences were then scanned for the presence or absence of each designed mutation codon. The list of mutations present in each variant was used to identify the abundance of each variant and the total number of unique variants sequenced. Average mutation load was calculated as the average across all unique variants. The presence or absence of each designed mutation was tallied for all variants in each sequencing run and used to calculate enrichment between naive library (preselection) and each selected run.

We calculated weighted mutation enrichment by comparing mutation counts to unmuted counts from each sequencing run. To calculate mutation frequency (*F*) in each run, amino acid mutation counts for each designed mutation were divided by the total counts for the wild-type and mutant amino acid at the gene position. The crude enrichment (*E*) was calculated by dividing the mutation frequency in a sequencing dataset from a sorted run by the equivalent mutation frequency observed in the preselection sequencing run. To calculate a weighted enrichment score (*WES*), the natural log of the crude enrichment was multiplied by the square root of the sum of the total mutation counts from both sorted and preselection sequencing sets. This weighted mutation enrichments for mutations that were highly sampled more heavily than mutations that were at very low frequencies in the library.

$$F=\frac{{Reads}_{mut}}{{Reads}_{mut}+{Reads}_{wt}}$$

$$E=\frac{F_{sort}}{F_{naïve\;}}$$

$$WES=\ln\left(E\right)\times \sqrt{{Reads}_{mut,sort}+{Reads}_{mut,naïve\;}}$$

### T cell culture

T cells were cultured in OpTimizer CTS T cell Expansion SFM containing 5% CTS Immune Cell SR (ThermoFisher, Waltham, MA), L-Glutamine, Penicillin/Streptomycin (Lonza, Basel, Switzerland), 10 mM N-Acetyl-L-cysteine (Sigma-Aldrich, St. Louis, MO), 300 IU/mL IL-2, 5 ng/mL IL-7, and 5 ng/mL IL-15 (PeproTech, Rocky Hill, NJ). T cells were activated with DynaBeads Human T-Activator CD3/CD28 (ThermoFisher, Waltham, MA) at a 2:1 bead:cell ratio for 48 h prior to electroporation. Following electroporation, T cells were re-stimulated with DynaBeads at a ratio of 1:2 (bead:cell) and maintained at ∼1E6 cells/mL.

### NK cell culture

NK cells were cultured in CTS AIM V SFM containing 5% CTS Immune cell SR (ThermoFisher, Waltham, MA), Penicillin/Streptomycin, and IL-2 (100 IU/mL). NK cells were activated by coculture with X-irradiated (100 Gy) feeder cells (K562 expressing membrane-bound IL-21 and 41BB-L)^83^ at indicated feeder:NK ratios (2:1 prior to electroporation, 5:1 48 h after electroporation, or 1:1 for all subsequent expansions).

### Electroporation of activated NK cells

Feeder cell-activated NK cells were washed once with PBS and resuspended at 3E7 cells/mL in electroporation buffer. Protector RNase inhibitor (Sigma-Aldrich, St. Louis, MO) was added to the mixture at a concentration of 0.8 U/μL and incubated for 5 min at room temperature. The cell mixture was added to 1 μg of transposase mRNA and 1 μg transposon nanoplasmid on ice. Transposon nanoplasmid alone was used as a control for all experiments. This mixture was electroporated in a 10-μL tip using the Neon Transfection System (ThermoFisher, Waltham, MA) under the following conditions: 1,850 V, pulse width of 10 ms, two pulses. NK cells were allowed to recover at a density of 1.5E6 cells/mL in antibiotic-free medium containing 1 μg/mL DNase I solution (STEMCELL Technologies, Cambridge, MA) for 30 min at 37°C, and were then cultured in complete NK cell medium at a density of 6E5 cells/mL. Forty-eight hours after electroporation, NK cells were expanded with feeder cells at a 5:1 feeder:NK ratio.

### T cell electroporation

After 48 h of stimulation, DynaBeads were magnetically removed, and T cells were washed once with PBS prior to resuspension in electroporation buffer. The 4D-Nucleofector (Lonza, Basel, Switzerland) and P3 kit was used with 1E6 T cells per 20-μL cuvette, 1 μg transposase mRNA, 1 μg transposon nanoplasmid, and the Nucleofector program FI-115 (optimized pipeline) or EO-142 (vector titration) was used. For all experiments, transposon nanoplasmid alone was used as a control for all experiments. T cells were allowed to recover in antibiotic-free medium containing 1 μg/mL DNase I solution (STEMCELL Technologies, Cambridge, MA) at 37°C, 5% CO_2_ for 30 min following gene transfer, and then were cultured in complete T cell medium and re-stimulated with DynaBeads. For cross electroporation platform comparisons, 1 μg transposase mRNA and 1.5 μg transposon nanoplasmid were used. Protocol and assemblies used for platforms were as follows: Amaxa 4D used program EO-142 in a 20-μL cuvette, MaxCyte (MaxCyte, Montgomery County, Maryland) used program T2 in an OC-100 2x 50-μL rxn assembly, and finally for the Neon Transfection System a 1,600-mV, 8-ms, 3-pulse electroporation was carried out in a 100-μL tip with R buffer. Post-electroporation recovery and subsequent restimulation was carried out as described above.

### NK cell intracellular cytokine staining assay

For intracellular cytokine staining, NK cells were plated at 2.5E6 cells/mL in NK cell medium without cytokines. After incubation overnight, the CD19+ Burkitt lymphoma cell line Raji was added at the indicated effector-to-target (E:T) ratios. Brilliant violet anti-CD107a was added to the culture and cells were incubated for 1 h at 37°C. Brefeldin A and monensin (BD Biosciences, San Jose, CA) were added and cells were incubated for an additional 4 h. Cells were stained with fixable viability dye, then for extracellular antigens. Cells were fixed and permeabilized using BD Cytofix/Cytoperm (BD Biosciences, San Jose, CA) following the manufacturer’s instructions. Cells were then stained for intracellular IFN-γ and TNF-⍺ and analyzed by flow cytometry.

### Target cell killing assays

T cells or NK cells were cultured overnight in medium without cytokines. Luciferase-expressing Raji cells were seeded into a black round-bottom 96-well plate (3E4-5E4 cells per well). T cells or NK cells were added to the wells in quadruplicate at the indicated E:T ratios. Target cells without effectors served as a negative control (spontaneous cell death) and target cells incubated with 1% Triton X-100 or NP40 served as a positive control (maximum killing). Co-cultures were incubated at 37°C for 24 h. After incubation, D-luciferin (potassium salt; Gold Biotechnology, St. Louis, MO) was added to each well at a final concentration of 25 μg/mL and incubated for 5 min with gentle shaking. Luminescence was read in endpoint mode using a BioTek Synergy microplate reader.

### Droplet-digital polymerase chain reaction

To assess the number of CD19-CAR-DHFR-eGFP construct copy numbers integrated into genomes, genomic DNA was first extracted from cells post engineering runs via GeneJET genomic DNA purification columns per manufacturer instructions (ThermoFisher). Junction PCR primers were designed using PrimerQuest software (Integrated DNA Technologies, Coralville, IA) using settings for two primers + probe qPCR (Table S5). Each sample was run as a duplexed assay consisting of a B2M internal reference primer + probe set (HEX) and an MND primer + probe set (FAM). Primers and probes were ordered from IDT. Reactions were set up using the ddPCR Supermix for Probes (no dUTP) (Bio-Rad, Hercules, CA) with 200 ng of genomic DNA per assay according to the manufacturer’s instructions. Droplets were generated and analyzed using the QX200 Droplet-digital PCR system (Bio-Rad, Hercules, CA). Frequency was calculated as fractional abundance adjusted for two copies of reference sequence per genome using the QuantaSoft version 14.0 software (Bio-Rad, Hercules, CA).

### Site-integration sequencing

Isolated genomic DNA from engineered CAR-T and CAR-NK cells from two donors was done as previously described.^72^ Briefly, sequencing libraries were prepared from 150 ng genomic DNA quantified by Picogreen (Life Technologies) using the Lotus DNA Library Prep Kit (Integrated DNA Technologies) according to the manufacturer’s specifications for libraries undergoing target enrichment. Ligations used vendor-supplied “stubby” adapters, with sample-specific 8-bp unique dual indices (UDIs) added during final library amplification. Hybridization capture was performed per manufacturer’s protocol with up to 16 libraries in multiplex (500 ng per library) using xGen universal blocking oligos (IDT) and a custom biotinylated xGen oligo probe pool designed to hybridize to the inserted transposon sequence. Given the small probe panel size, hybridizations and temperature-sensitive washes were performed at 63°C and the total hybridization time was increased from 4 h to 16 h. Captured libraries were then amplified to ≥2 nM using KAPA HiFi HotStart 2X PCR master mix, quantified by Picogreen, sized on an Agilent TapeStation using the D1000 assay, normalized, and pooled for 150-bp paired-end sequencing on an Illumina NovaSeq∗ SP flowcell. Custom probe sequences are reported in Table S1.

### Site-integration analysis

We analyzed integration-site data for TcBuster and compared results with published literature data for integration sites of other transposase and viral systems as previously described.^72^ Specifically, comparison sequencing datasets were generated by outside sources using different experimental methods. Raw reads from comparison datasets were retrieved (Accession IDs: Lentivirus: SAMN11351981, SAMN11351982, SAMN11351983, SAMN11351984, SAMN00188192, SAMN00188193; Sleeping Beauty: SAMN02870102; and PiggyBac: SAMN02870101) and computationally mapped and analyzed in the same manner as the in-house generated data using a custom Python script. Merged reads were filtered to exclude reads containing only transposon vector sequences (bbmap v39.03^84^) and to isolate reads spanning a transposon-genome junction (cutadapt v4.6^85^). We then mapped the genomic portion of the junction reads (Bowtie2 v2.4.4^86^). We mapped the reads to the RefSeq genome annotations (retrieved from Karolchik et al.^87^).

### *In vivo* study design

Specific pathogen-free female NOD-*scid* IL2Rgamma^null^ (NSG) mice were purchased from The Jackson Laboratory (RRID:IMSR_JAX:005557). Tumor challenge studies were performed using the Raji-luc cell line, a well-defined model of Burkitt lymphoma used for testing CAR-T function. Specifically, mice were implanted with 1E5 Raji-luc cells resuspended in PBS and delivered in 100 μL via tail vein. Three days post tumor implantation, the mice were randomized into treatment groups (*n* = 6–10) and received either PBS, 5E6 CAR-NK from two donors as indicated, or donor-matched non-engineered NK in 100 μL PBS via tail vein injection.

For CAR-T studies, mice were challenged with 1E5 Raji-luc cells and given either 1E5 CAR-T (low) or 1E6 CAR-T (high) at 6 days post tumor challenge, cells were engineered from two donors. All tumor growth was monitored by weekly bioluminescence imaging of mice 5 min post intraperitoneal injection of D-luciferin (100 μL total volume, 28 mg/kg) using an IVIS100 imager followed by region of interest analysis of tumor images (Living Image software, version 4.7.3). At endpoint, spleen and bone marrow were collected and processed to single-cell suspensions through standard mashing and ACK-processing or as previously described^88^ and examined for remaining CAR-construct expressing cells. This study was carried out in strict accordance with the recommendations in the Guide for the Care and Use of Laboratory Animals of the National Institutes of Health. The protocol and all procedures were approved by the University of Minnesota Institutional Animal Care and Use Committee (Protocol 1905-37099A). The health of the mice was monitored daily by University of Minnesota veterinary staff.

### Statistical analysis

The Student’s t test was used to evaluate the significance of differences between the two groups. Differences between three or more groups with one data point were evaluated by a one-way ANOVA test. Differences among three or more groups with multiple data points were evaluated by a two-way ANOVA test. Differences among three or more groups with multiple data points were evaluated by the Log rank (Mantel-Cox) test. All assays were repeated with at least two independent donors. The level of significance was set at α = 0.05 and corrected for multiple comparisons when applicable. All statistical analyses were performed using GraphPad Prism 10.1.1.

## Data and code availability

The data underlying this article will be shared on reasonable request to the corresponding author.

## Supplemental information

Document S1. Figures S1–S10 and Tables S1–S5

Document S2. Article plus supplemental information
