# Supplementary material for: Evolution of the clinical-stage hyperactive TcBuster transposase as a platform for robust non-viral production of adoptive cellular therapies
Source: Mol Ther. 2024 Apr 16;32(6):1817–34. doi: 10.1016/j.ymthe.2024.04.024 (PMC11184336; doi:10.1016/j.ymthe.2024.04.024)
Supplement: Document S1. Figures S1–S10 and Tables S1–S5 [file mmc1.pdf]

YMTHE, Volume *32*

Supplemental Information

Evolution of the clinical-stage hyperactive TcBuster transposase as a platform for robust non-viral production of adoptive cellular therapies

Joseph G. Skeate, Emily J. Pomeroy, Nicholas J. Slipek, Bryan J. Jones, Bryce J. Wick, Jae-Woong Chang, Walker S. Lahr, Erin M. Stelljes, Xiaobai Patrinostro, Blake Barnes, Trevor Zarecki, Joshua B. Krueger, Jacob E. Bridge, Gabrielle M. Robbins, Madeline D. McCormick, John R. Leerar, Kari T. Wenzel, Kathlyn M. Hornberger, Kirsti Walker, Dalton Smedley, David A. Largaespada, Neil Otto, Beau R. Webber, and Branden S. Moriarity

Table S1. Primer and probe list for site-integration sequencing.

Chromosome	Start	Stop	Name	Score	Strand	Sequence	GC Percent	Length
	0	120	778612_43767617_5_Common_TCB_1_1	0		CAGTGTTCTTCAACCTTTGCCATCCGGCGGAACCCTTTGTCGAGATATTTTTTTTTATGGAACCCTTCATTTAGTAATACACCCAGATGAGATTTTAGGGACAGCTGCGTTGACTTGTTA	42	120
	10	130	778612_43767617_5_Common_TCB_1_2	0		CAACCTTTGCCATCCGGCGGAACCCTTTGTCGAGATATTTTTTTTTATGGAACCCTTCATTTAGTAATACACCCAGATGAGATTTTAGGGACAGCTGCGTTGACTTGTTACGAACAAGGT	42	120
	20	140	778612_43767617_5_Common_TCB_1_3	0		CATCCGGCGGAACCCTTTGTCGAGATATTTTTTTTTATGGAACCCTTCATTTAGTAATACACCCAGATGAGATTTTAGGGACAGCTGCGTTGACTTGTTACGAACAAGGTGAGCCCGTGC	45	120
	30	150	778612_43767617_5_Common_TCB_1_4	0		AACCCTTTGTCGAGATATTTTTTTTTATGGAACCCTTCATTTAGTAATACACCCAGATGAGATTTTAGGGACAGCTGCGTTGACTTGTTACGAACAAGGTGAGCCCGTGCTTTGGTAATA	40	120
	40	160	778612_43767617_5_Common_TCB_1_5	0		CGAGATATTTTTTTTTATGGAACCCTTCATTTAGTAATACACCCAGATGAGATTTTAGGGACAGCTGCGTTGACTTGTTACGAACAAGGTGAGCCCGTGCTTTGGTAATAAAAACTCTAA	38	120
	50	170	778612_43767617_5_Common_TCB_1_6	0		TTTTTTATGGAACCCTTCATTTAGTAATACACCCAGATGAGATTTTAGGGACAGCTGCGTTGACTTGTTACGAACAAGGTGAGCCCGTGCTTTGGTAATAAAAACTCTAAATAAGATTTA	37	120
	60	180	778612_43767617_5_Common_TCB_1_7	0		AACCCTTCATTTAGTAATACACCCAGATGAGATTTTAGGGACAGCTGCGTTGACTTGTTACGAACAAGGTGAGCCCGTGCTTTGGTAATAAAAACTCTAAATAAGATTTAAATTTGCATT	37	120
	70	190	778612_43767617_5_Common_TCB_1_8	0		TTAGTAATACACCCAGATGAGATTTTAGGGACAGCTGCGTTGACTTGTTACGAACAAGGTGAGCCCGTGCTTTGGTAATAAAAACTCTAAATAAGATTTAAATTTGCATTTATTTAAACA	34	120
	80	200	778612_43767617_5_Common_TCB_1_9	0		ACCCAGATGAGATTTTAGGGACAGCTGCGTTGACTTGTTACGAACAAGGTGAGCCCGTGCTTTGGTAATAAAAACTCTAAATAAGATTTAAATTTGCATTTATTTAAACAAACTTTAAAC	34	120
	90	210	778612_43767617_5_Common_TCB_1_10	0		GATTTTAGGGACAGCTGCGTTGACTTGTTACGAACAAGGTGAGCCCGTGCTTTGGTAATAAAAACTCTAAATAAGATTTAAATTTGCATTTATTTAAACAAACTTTAAACAAAAAGATAA	31	120
	100	220	778612_43767617_5_Common_TCB_1_11	0		ACAGCTGCGTTGACTTGTTACGAACAAGGTGAGCCCGTGCTTTGGTAATAAAAACTCTAAATAAGATTTAAATTTGCATTTATTTAAACAAACTTTAAACAAAAAGATAAATATTCCAAA	29	120
	110	230	778612_43767617_5_Common_TCB_1_12	0		TGACTTGTTACGAACAAGGTGAGCCCGTGCTTTGGTAATAAAAACTCTAAATAAGATTTAAATTTGCATTTATTTAAACAAACTTTAAACAAAAAGATAAATATTCCAAATAAAATAATA	24	120
	120	240	778612_43767617_5_Common_TCB_1_13	0		CGAACAAGGTGAGCCCGTGCTTTGGTAATAAAAACTCTAAATAAGATTTAAATTTGCATTTATTTAAACAAACTTTAAACAAAAAGATAAATATTCCAAATAAAATAATATATAAAATAA	22	120
	130	250	778612_43767617_5_Common_TCB_1_14	0		GAGCCCGTGCTTTGGTAATAAAAACTCTAAATAAGATTTAAATTTGCATTTATTTAAACAAACTTTAAACAAAAAGATAAATATTCCAAATAAAATAATATATAAAATAAAAAATAAAAA	18	120
	140	260	778612_43767617_5_Common_TCB_1_15	0		TTTGGTAATAAAAACTCTAAATAAGATTTAAATTTGCATTTATTTAAACAAACTTTAAACAAAAAGATAAATATTCCAAATAAAATAATATATAAAATAAAAAATAAAAATTAGATCCAG	14	120
	150	270	778612_43767617_5_Common_TCB_1_16	0		AAAACTCTAAATAAGATTTAAATTTGCATTTATTTAAACAAACTTTAAACAAAAAGATAAATATTCCAAATAAAATAATATATAAAATAAAAAATAAAAATTAGATCCAGACATGTAGGT	16	120
	160	280	778612_43767617_5_Common_TCB_1_17	0		ATAAGATTTAAATTTGCATTTATTTAAACAAACTTTAAACAAAAAGATAAATATTCCAAATAAAATAATATATAAAATAAAAAATAAAAATTAGATCCAGACATGTAGGTCTTGAAAGGA	18	120
	170	290	778612_43767617_5_Common_TCB_1_18	0		AATTTGCATTTATTTAAACAAACTTTAAACAAAAAGATAAATATTCCAAATAAAATAATATATAAAATAAAAAATAAAAATTAGATCCAGACATGTAGGTCTTGAAAGGAGTGGGAATTG	21	120
	180	300	778612_43767617_5_Common_TCB_1_19	0		TATTTAAACAAACTTTAAACAAAAAGATAAATATTCCAAATAAAATAATATATAAAATAAAAAATAAAAATTAGATCCAGACATGTAGGTCTTGAAAGGAGTGGGAATTGGCTCCGGTGC	26	120
	190	310	778612_43767617_5_Common_TCB_1_20	0		AACTTTAAACAAAAAGATAAATATTCCAAATAAAATAATATATAAAATAAAAAATAAAAATTAGATCCAGACATGTAGGTCTTGAAAGGAGTGGGAATTGGCTCCGGTGCGGATCCGGGA	31	120
	200	313	778612_43767617_5_Common_TCB_1_21	0		AAAAAGATAAATATTCCAAATAAAATAATATATAAAATAAAAAATAAAAATTAGATCCAGACATGTAGGTCTTGAAAGGAGTGGGAATTGGCTCCGGTGCGGATCCGGGATCC	33	113
	0	120	778612_43767618_3_Common_TCB_1_1	0		GCCGAGAGACGGAAGAACCGCTTGGCGTAATCCCTGCAGGGATCCCATTGTCTGAGTAGGTGTCATTCTATTCTGGGGGGTGGGGTGGGGCAGGACAGCAAGGGGGAGGATTGGGAAGAC	59	120
	10	130	778612_43767618_3_Common_TCB_1_2	0		GGAAGAACCGCTTGGCGTAATCCCTGCAGGGATCCCATTGTCTGAGTAGGTGTCATTCTATTCTGGGGGGTGGGGTGGGGCAGGACAGCAAGGGGGAGGATTGGGAAGACAATAGCAGGC	57	120
	20	140	778612_43767618_3_Common_TCB_1_3	0		CTTGGCGTAATCCCTGCAGGGATCCCATTGTCTGAGTAGGTGTCATTCTATTCTGGGGGGTGGGGTGGGGCAGGACAGCAAGGGGGAGGATTGGGAAGACAATAGCAGGCAGAATTCATT	54	120
	30	150	778612_43767618_3_Common_TCB_1_4	0		TCCCTGCAGGGATCCCATTGTCTGAGTAGGTGTCATTCTATTCTGGGGGGTGGGGTGGGGCAGGACAGCAAGGGGGAGGATTGGGAAGACAATAGCAGGCAGAATTCATTTCTGAACGAT	53	120
	40	160	778612_43767618_3_Common_TCB_1_5	0		GATCCCATTGTCTGAGTAGGTGTCATTCTATTCTGGGGGGTGGGGTGGGGCAGGACAGCAAGGGGGAGGATTGGGAAGACAATAGCAGGCAGAATTCATTTCTGAACGATTCTAGGTTAG	51	120
	50	170	778612_43767618_3_Common_TCB_1_6	0		TCTGAGTAGGTGTCATTCTATTCTGGGGGGTGGGGTGGGGCAGGACAGCAAGGGGGAGGATTGGGAAGACAATAGCAGGCAGAATTCATTTCTGAACGATTCTAGGTTAGGATCAAACAA	49	120
	60	180	778612_43767618_3_Common_TCB_1_7	0		TGTCATTCTATTCTGGGGGGTGGGGTGGGGCAGGACAGCAAGGGGGAGGATTGGGAAGACAATAGCAGGCAGAATTCATTTCTGAACGATTCTAGGTTAGGATCAAACAAAATACAATTT	46	120
	70	190	778612_43767618_3_Common_TCB_1_8	0		TTCTGGGGGGTGGGGTGGGGCAGGACAGCAAGGGGGAGGATTGGGAAGACAATAGCAGGCAGAATTCATTTCTGAACGATTCTAGGTTAGGATCAAACAAAATACAATTTATTTTAAAAC	44	120
	80	200	778612_43767618_3_Common_TCB_1_9	0		TGGGGTGGGGCAGGACAGCAAGGGGGAGGATTGGGAAGACAATAGCAGGCAGAATTCATTTCTGAACGATTCTAGGTTAGGATCAAACAAAATACAATTTATTTTAAAACTGTAAGTTAA	40	120
	90	210	778612_43767618_3_Common_TCB_1_10	0		CAGGACAGCAAGGGGGAGGATTGGGAAGACAATAGCAGGCAGAATTCATTTCTGAACGATTCTAGGTTAGGATCAAACAAAATACAATTTATTTTAAAACTGTAAGTTAACTTACCTTTG	37	120
	100	220	778612_43767618_3_Common_TCB_1_11	0		AGGGGGAGGATTGGGAAGACAATAGCAGGCAGAATTCATTTCTGAACGATTCTAGGTTAGGATCAAACAAAATACAATTTATTTTAAAACTGTAAGTTAACTTACCTTTGCTTGTCTAAA	34	120
	110	230	778612_43767618_3_Common_TCB_1_12	0		TTGGGAAGACAATAGCAGGCAGAATTCATTTCTGAACGATTCTAGGTTAGGATCAAACAAAATACAATTTATTTTAAAACTGTAAGTTAACTTACCTTTGCTTGTCTAAACCTAAAACAA	31	120
	120	240	778612_43767618_3_Common_TCB_1_13	0		AATAGCAGGCAGAATTCATTTCTGAACGATTCTAGGTTAGGATCAAACAAAATACAATTTATTTTAAAACTGTAAGTTAACTTACCTTTGCTTGTCTAAACCTAAAACAACAACAAAACT	29	120
	130	250	778612_43767618_3_Common_TCB_1_14	0		AGAATTCATTTCTGAACGATTCTAGGTTAGGATCAAACAAAATACAATTTATTTTAAAACTGTAAGTTAACTTACCTTTGCTTGTCTAAACCTAAAACAACAACAAAACTACGACCACAA	29	120
	140	260	778612_43767618_3_Common_TCB_1_15	0		TCTGAACGATTCTAGGTTAGGATCAAACAAAATACAATTTATTTTAAAACTGTAAGTTAACTTACCTTTGCTTGTCTAAACCTAAAACAACAACAAAACTACGACCACAAGTACAGTTAC	31	120
	150	270	778612_43767618_3_Common_TCB_1_16	0		TCTAGGTTAGGATCAAACAAAATACAATTTATTTTAAAACTGTAAGTTAACTTACCTTTGCTTGTCTAAACCTAAAACAACAACAAAACTACGACCACAAGTACAGTTACATATTTTTGA	28	120
	160	280	778612_43767618_3_Common_TCB_1_17	0		GATCAAACAAAATACAATTTATTTTAAAACTGTAAGTTAACTTACCTTTGCTTGTCTAAACCTAAAACAACAACAAAACTACGACCACAAGTACAGTTACATATTTTTGAAAATTAAGGT	27	120
	170	290	778612_43767618_3_Common_TCB_1_18	0		AATACAATTTATTTTAAAACTGTAAGTTAACTTACCTTTGCTTGTCTAAACCTAAAACAACAACAAAACTACGACCACAAGTACAGTTACATATTTTTGAAAATTAAGGTTAAGTGCAGT	28	120
	180	300	778612_43767618_3_Common_TCB_1_19	0		ATTTTAAAACTGTAAGTTAACTTACCTTTGCTTGTCTAAACCTAAAACAACAACAAAACTACGACCACAAGTACAGTTACATATTTTTGAAAATTAAGGTTAAGTGCAGTGTAAGTCAAC	30	120
	190	310	778612_43767618_3_Common_TCB_1_20	0		TGTAAGTTAACTTACCTTTGCTTGTCTAAACCTAAAACAACAACAAAACTACGACCACAAGTACAGTTACATATTTTTGAAAATTAAGGTTAAGTGCAGTGTAAGTCAACTATGCGAATG	32	120
	200	320	778612_43767618_3_Common_TCB_1_21	0		CTTACCTTTGCTTGTCTAAACCTAAAACAACAACAAAACTACGACCACAAGTACAGTTACATATTTTTGAAAATTAAGGTTAAGTGCAGTGTAAGTCAACTATGCGAATGGATAACATGT	33	120
	210	330	778612_43767618_3_Common_TCB_1_22	0		CTTGTCTAAACCTAAAACAACAACAAAACTACGACCACAAGTACAGTTACATATTTTTGAAAATTAAGGTTAAGTGCAGTGTAAGTCAACTATGCGAATGGATAACATGTTTCAACATGA	32	120
	220	340	778612_43767618_3_Common_TCB_1_23	0		CCTAAAACAACAACAAAACTACGACCACAAGTACAGTTACATATTTTTGAAAATTAAGGTTAAGTGCAGTGTAAGTCAACTATGCGAATGGATAACATGTTTCAACATGAAACTCCGATT	33	120
	230	350	778612_43767618_3_Common_TCB_1_24	0		CAACAAAACTACGACCACAAGTACAGTTACATATTTTTGAAAATTAAGGTTAAGTGCAGTGTAAGTCAACTATGCGAATGGATAACATGTTTCAACATGAAACTCCGATTGACGCATGTG	36	120
	240	360	778612_43767618_3_Common_TCB_1_25	0		ACGACCACAAGTACAGTTACATATTTTTGAAAATTAAGGTTAAGTGCAGTGTAAGTCAACTATGCGAATGGATAACATGTTTCAACATGAAACTCCGATTGACGCATGTGCATTCTGAAG	37	120
	250	370	778612_43767618_3_Common_TCB_1_26	0		GTACAGTTACATATTTTTGAAAATTAAGGTTAAGTGCAGTGTAAGTCAACTATGCGAATGGATAACATGTTTCAACATGAAACTCCGATTGACGCATGTGCATTCTGAAGAGCGGCGCGG	40	120
	260	380	778612_43767618_3_Common_TCB_1_27	0		ATATTTTTGAAAATTAAGGTTAAGTGCAGTGTAAGTCAACTATGCGAATGGATAACATGTTTCAACATGAAACTCCGATTGACGCATGTGCATTCTGAAGAGCGGCGCGGCCGACGTCTC	42	120
	270	390	778612_43767618_3_Common_TCB_1_28	0		AAATTAAGGTTAAGTGCAGTGTAAGTCAACTATGCGAATGGATAACATGTTTCAACATGAAACTCCGATTGACGCATGTGCATTCTGAAGAGCGGCGCGGCCGACGTCTCTCGAATTGAA	44	120
	280	400	778612_43767618_3_Common_TCB_1_29	0		TAAGTGCAGTGTAAGTCAACTATGCGAATGGATAACATGTTTCAACATGAAACTCCGATTGACGCATGTGCATTCTGAAGAGCGGCGCGGCCGACGTCTCTCGAATTGAAGCAATGACTC	47	120
	290	410	778612_43767618_3_Common_TCB_1_30	0		GTAAGTCAACTATGCGAATGGATAACATGTTTCAACATGAAACTCCGATTGACGCATGTGCATTCTGAAGAGCGGCGCGGCCGACGTCTCTCGAATTGAAGCAATGACTCGCGGAACCCC	50	120
	300	420	778612_43767618_3_Common_TCB_1_31	0		TATGCGAATGGATAACATGTTTCAACATGAAACTCCGATTGACGCATGTGCATTCTGAAGAGCGGCGCGGCCGACGTCTCTCGAATTGAAGCAATGACTCGCGGAACCCCGAAAGCCTTT	50	120
	310	430	778612_43767618_3_Common_TCB_1_32	0		GATAACATGTTTCAACATGAAACTCCGATTGACGCATGTGCATTCTGAAGAGCGGCGCGGCCGACGTCTCTCGAATTGAAGCAATGACTCGCGGAACCCCGAAAGCCTTTGGGTGGAACC	52	120
	320	440	778612_43767618_3_Common_TCB_1_33	0		TTCAACATGAAACTCCGATTGACGCATGTGCATTCTGAAGAGCGGCGCGGCCGACGTCTCTCGAATTGAAGCAATGACTCGCGGAACCCCGAAAGCCTTTGGGTGGAACCCTAGGGTTCC	55	120
	330	450	778612_43767618_3_Common_TCB_1_34	0		AACTCCGATTGACGCATGTGCATTCTGAAGAGCGGCGCGGCCGACGTCTCTCGAATTGAAGCAATGACTCGCGGAACCCCGAAAGCCTTTGGGTGGAACCCTAGGGTTCCGCGGAACACA	57	120
	340	460	778612_43767618_3_Common_TCB_1_35	0		GACGCATGTGCATTCTGAAGAGCGGCGCGGCCGACGTCTCTCGAATTGAAGCAATGACTCGCGGAACCCCGAAAGCCTTTGGGTGGAACCCTAGGGTTCCGCGGAACACAGGTTGAAGAA	57	120
	350	465	778612_43767618_3_Common_TCB_1_36	0		CATTCTGAAGAGCGGCGCGGCCGACGTCTCTCGAATTGAAGCAATGACTCGCGGAACCCCGAAAGCCTTTGGGTGGAACCCTAGGGTTCCGCGGAACACAGGTTGAAGAACACTG	57	115

Table S2. Summary data from Site-integration sequencing

Name	Total unique insertion sites	Insertion sites not in transcript (%)	Insertion sites within transcript (%)	Insertion sites within intron (%)	Insertion sites within exon (%)	Insertion sites within coding exon (%)	Median distance to transcriptional start site (kB)	Most abundant clone reads (%)	Top 10 most abundant clone reads (%)
NKLP2_CAR_NK	5,653	43.0%	57.0%	53.3%	6.4%	0.8%	20.22	0.236%	1.916%
NKLP3_CAR_NK	4,397	40.8%	59.2%	53.4%	8.4%	1.2%	19.28	0.497%	2.875%
NKLP2_CAR_T	15,991	36.8%	63.2%	59.0%	7.0%	1.1%	16.38	0.061%	0.447%
NKLP3_CAR_T	19,673	36.3%	63.7%	59.7%	6.7%	1.2%	16.21	0.046%	0.370%
lenti (Wang)	27,482 ±990	15.2% ±0.2%	84.8% ±0.2%	79.9% ±0.1%	7.8% ±0.1%	3.1% ±0.2%	14.0 ±0.3	0.502% ±0.248%	0.963% ±0.345%
lenti (Brady)	266 ±48	15.6% ±0.9%	84.4% ±0.9%	80.2% ±2.1%	8.% ±3.9%	3.2% ±1.5%	11.2 ±2.2	2.237% ±0.565%	13.136% ±1.777%
PiggyBac	22,604 ±21,446	42.2% ±3.6%	57.8% ±3.6%	55.8% ±2.9%	3.7% ±1.8%	0.4% ±0.3%	23.6 ±6.1	2.517% ±1.744%	9.119% ±2.83%
Sleeping Beauty	34,206 ±20,319	49.7% ±4.5%	50.3% ±4.5%	49.3% ±4.2%	1.7% ±0.6%	0.1% ±0.1%	33.8 ±5.1	2.376% ±1.37%	9.958% ±7.764%
Random	72,961 ±33,541	46.3% ±0.3%	53.8% ±0.3%	51.2% ±0.3%	4.3% ±0.%	1.3% ±0.%	29.6 ±0.3	0.002% ±0.%	0.015% ±0.008%

Table S3. Sorted cell counts upstream NGS analysis

Condition	brightness	Cell count
pre-sort	n/a	Lots (not sorted)
50ng	negative	178,280
50ng	Double positive	44,096
50ng	bright	1,900
500ng	negative	323,509
500ng	Double positive	405,962
500ng	bright	302,312

Table S4. Next generation sequencing primer list

o2510	5' TcB Shuffling oligo	TCAATGGGATTTCTGGGCCTGTTTGTTGTTGCACAGGTTGTCAATGTCGG
o2541	TcBFHomolKazAgel	TTTGACCTCCATAGAAGACACCGACTCTAGAGGATCCACCGGTGCCACCATGATGCTGAATTGGCTGAAGAGC
o2542	TcBRHomolAgeI	ATTATGATCTAGAGTCGCGGCCAAACTCAATGGGGATACCGGTTCAATGGGATTTCTGGGCCTG
o271	End-SV40-FWD	GGTTCAGGGGGAGGTGTG
O895<br><br>Splice Acceptor	MND-1 R	CCAATCAGTTCGCTTCTCGC<br><br>ATCGATCGCAGGCGCAATCTTCGCATTTCTTTTTTCCAG

Table S5. Primer and probe list for ddPCR analysis

Primer or Probe Set	Sequence
MND F Primer	CTGAAATGACCCTGTGCCTTAT
MND R Primer	GCGATCTGACGGTTCACTAAA
MND Probe	/56-FAM/ACCAATCAG/ZEN/TTCGCTTCTCGCTTCT/3IABkFQ/
B2M Ex3 F Primer	AGATTTGGACCTGCGAGCG
B2M Ex3 R Primer	GAGCGGCTGTCTCCACAAGT
B2M Ex3 Probe	/5HEX/TTCTGACCT/ZEN/GAAGGCTCTGCGCG/3IABkFQ/

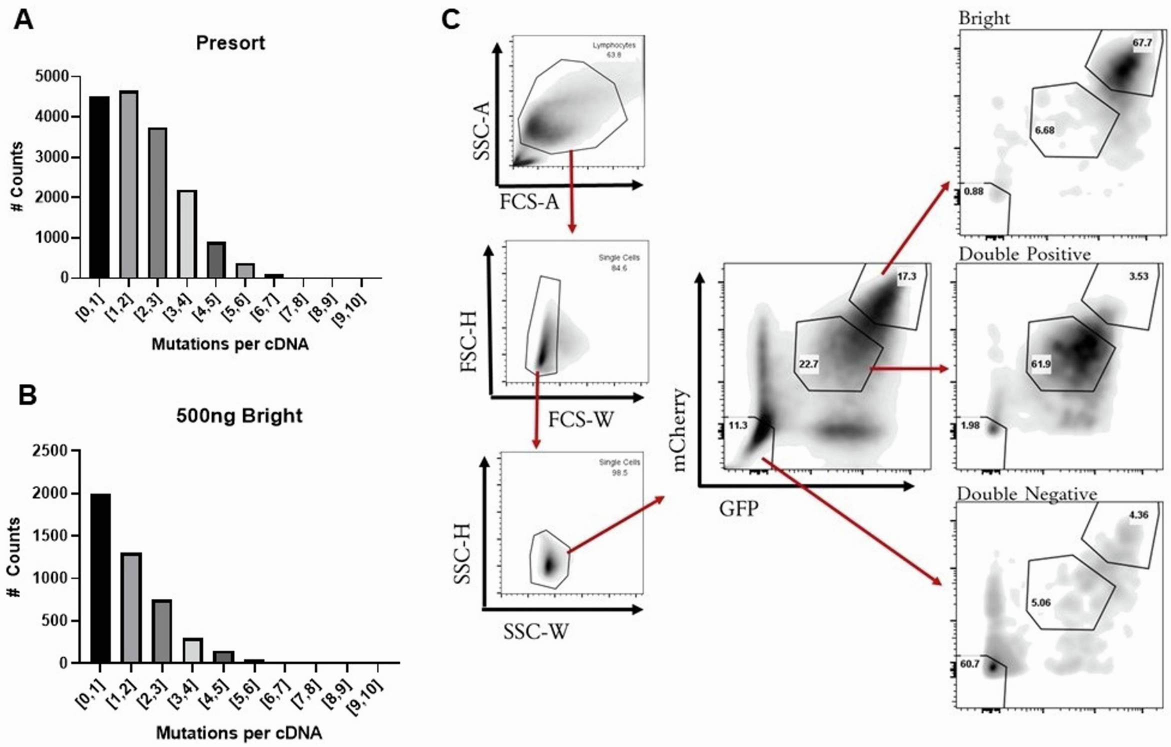

Figure S1. Mutation Frequency for Presort and Postsort Libraries and FACS Gating for High-Throughput Screening Platform. (A, B) Number of sequencing reads (counts) for the different mutation loads per cDNA for Presorted and 500ng Superbright postsort libraries. (C) Gating strategy used to isolate cells that 1) highly express both mCherry and GFP (Bright) 2) express both mCherry and GFP and moderate levels (Double Positive) 3) Contained neither mCherry or GFP but were hygromycin resistant (Double Negative). Isolated populations were subsequently sent to NGS for analysis.

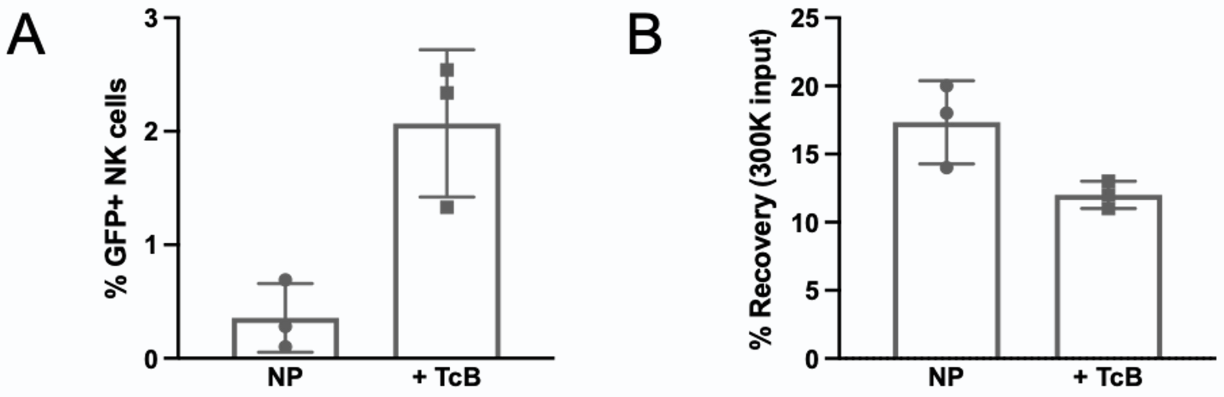

Figure S2. Transposition efficiency and cell health are poor when cells are electroporated on day 7 of activation. Primary human peripheral blood (PB) NK cells were expanded for 7 days with mbIL21- and 41BBL-expressing K562 feeder cells at a 2:1 (feeder:NK) ratio. NK cells were electroporated with the nanoplasmid transposon (NP) alone or in combination with mRNA encoding *TcBuster* (TcB). Two days after electroporation, NK cells were expanded with feeder cells (1:1 feeder:NK ratio) for 1 week to allow for the loss of transient NP expression. After this expansion, GFP expression was measured by flow cytometry (A) and cells were counted to calculate recovery from electroporation input (B).

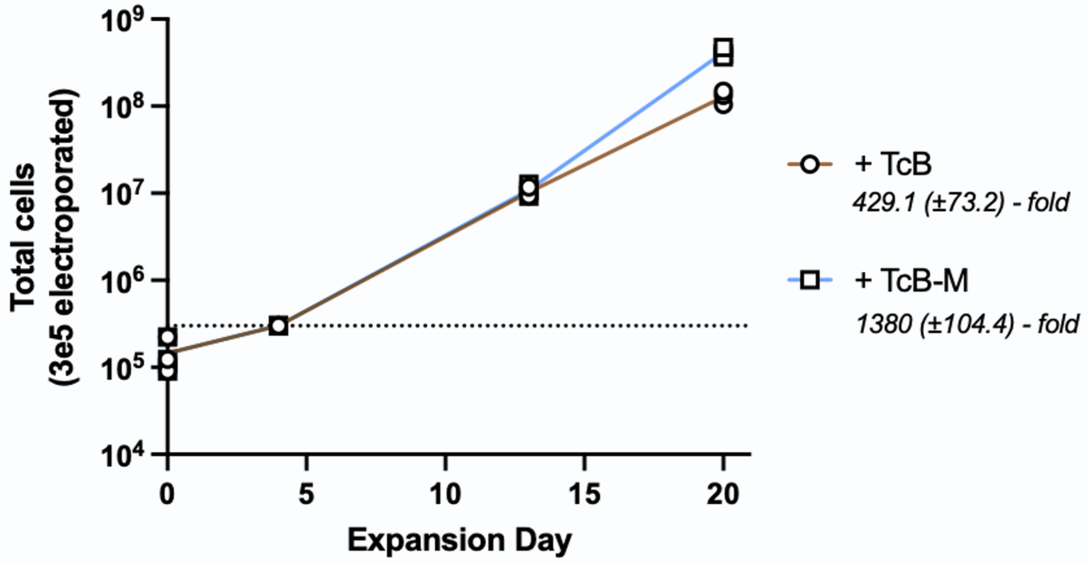

Figure S3. Fold expansion of CAR-NK cells from electroporation input during engineering pipeline. Cells were quantified through countess II using trypan blue exclusion.

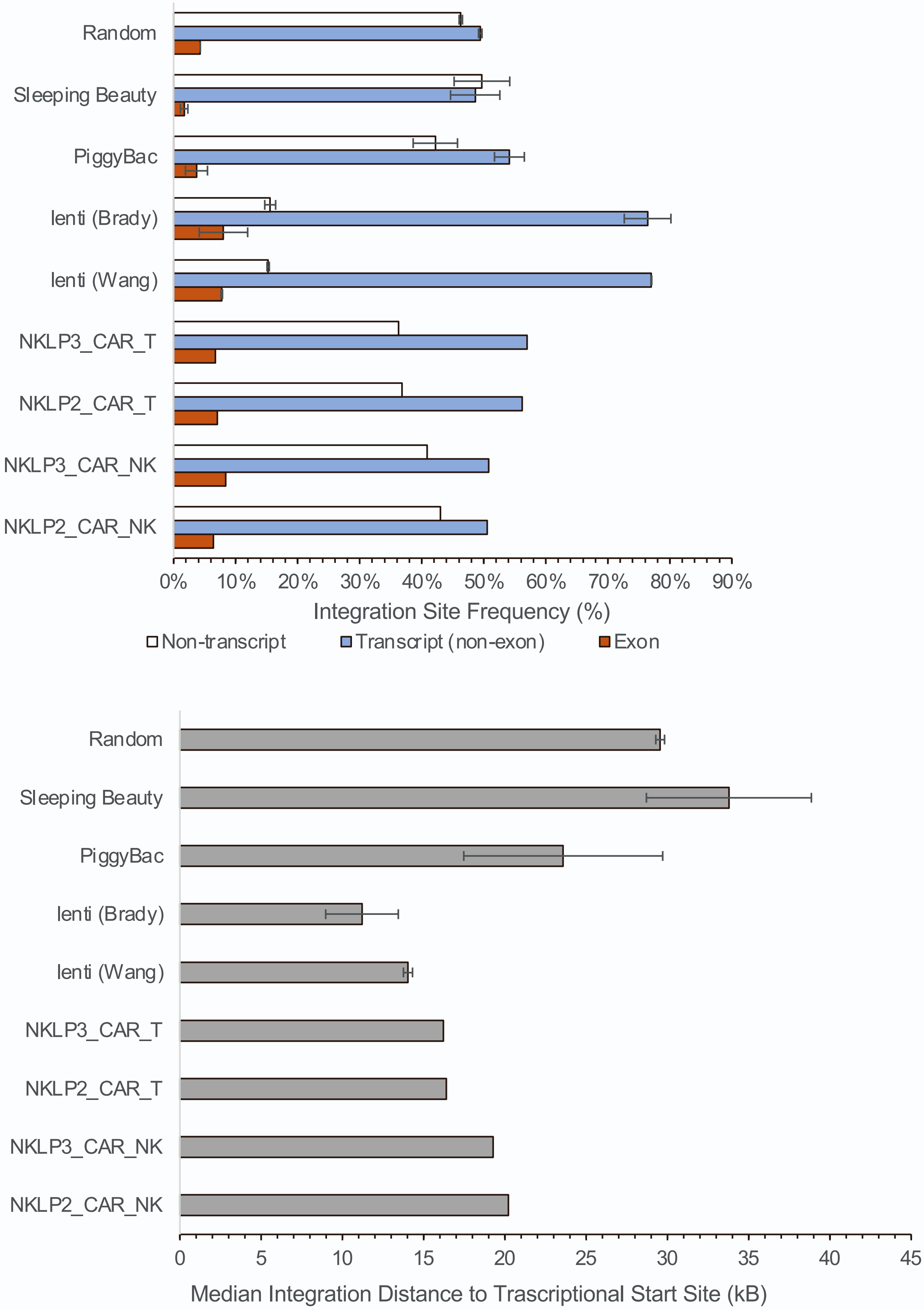

Figure S4 – Site-integration sequencing results of CD19-CAR-DHFR-eGFP construct in primary cells within this study.

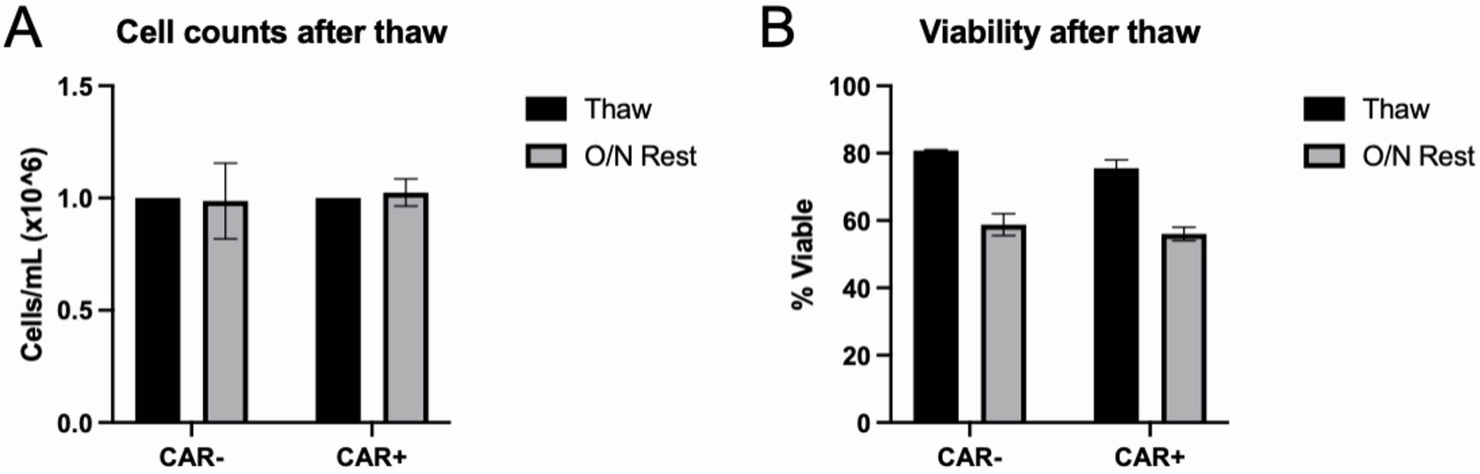

Figure S5 - NK cell viability and recovery after cryopreservation. CAR-positive and CAR-negative NK cells were cryopreserved after the 20-day production timeline. After thaw, cells were counted (A), and viability was measured by trypan blue exclusion (B). Cells were immediately plated in killing assays or plated at a density of 1E6 cells/mL in media containing 100 IU/mL IL2 overnight. After overnight rest, cells were counted, and viability was measured again.

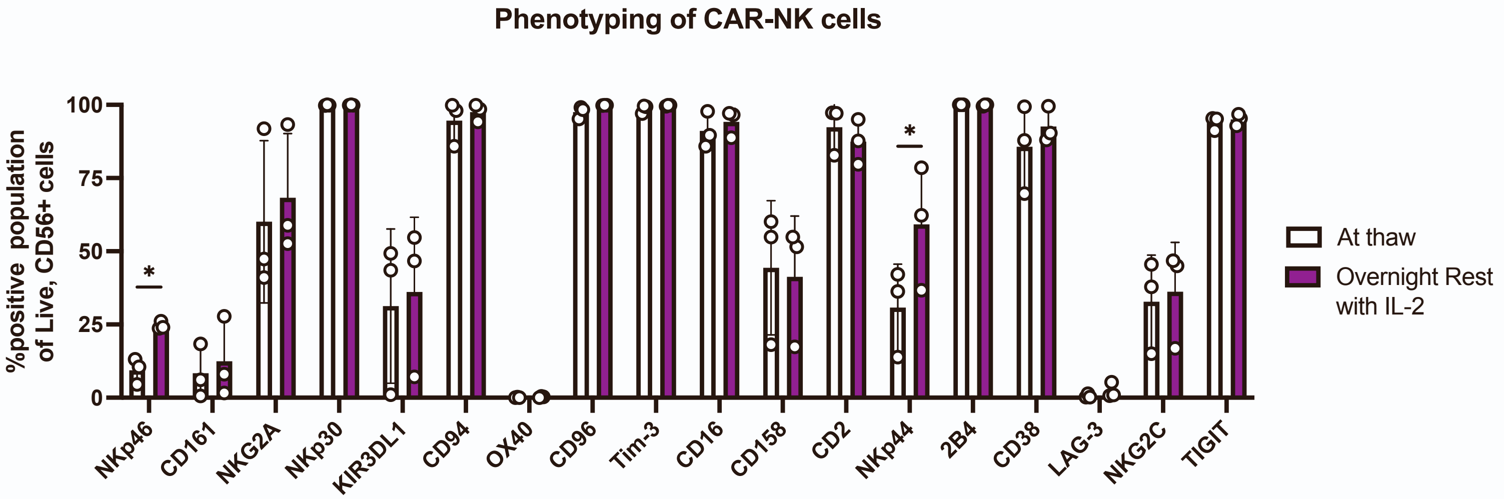

Figure S6 – General phenotyping of CAR-NK cells at thaw and post overnight rest in IL-2 media show increases in NKp46 and NKp44. CAR-NK cells were phenotyped via flow cytometry as Live, CD56+, GFP+ cells and sub-gated for indicated marker positivity immediately post thaw from cryopreservation and post 14h overnight rest in IL-2 containing media. Three independent donors shown. **p<0.05, paired student's t-test*

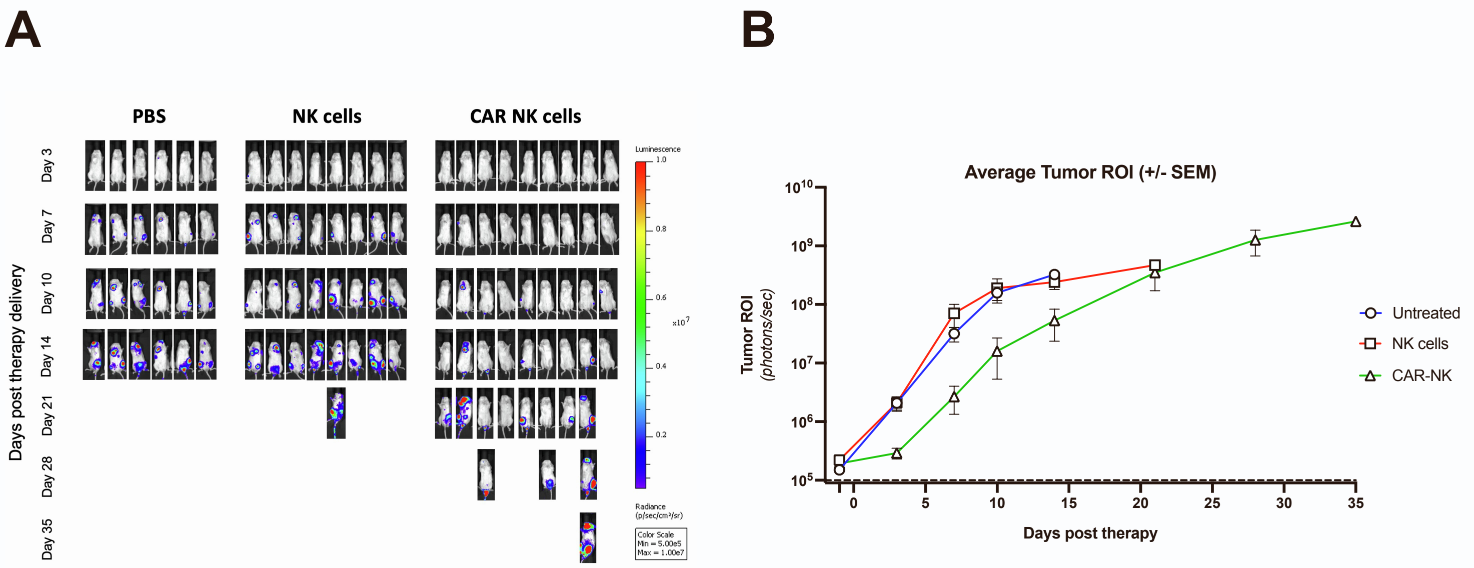

Figure S7 – BLI images and average tumor ROIs from all groups in study receiving NK cell therapy. (A) BLI images from Raji challenged mice receiving PBS (6), 5E6 NK cells (8, 2 donors), or 5E6 CAR-NK cells (9, 2 donors). **(B)** Average tumor ROI plotted from all groups (+/- SEM).

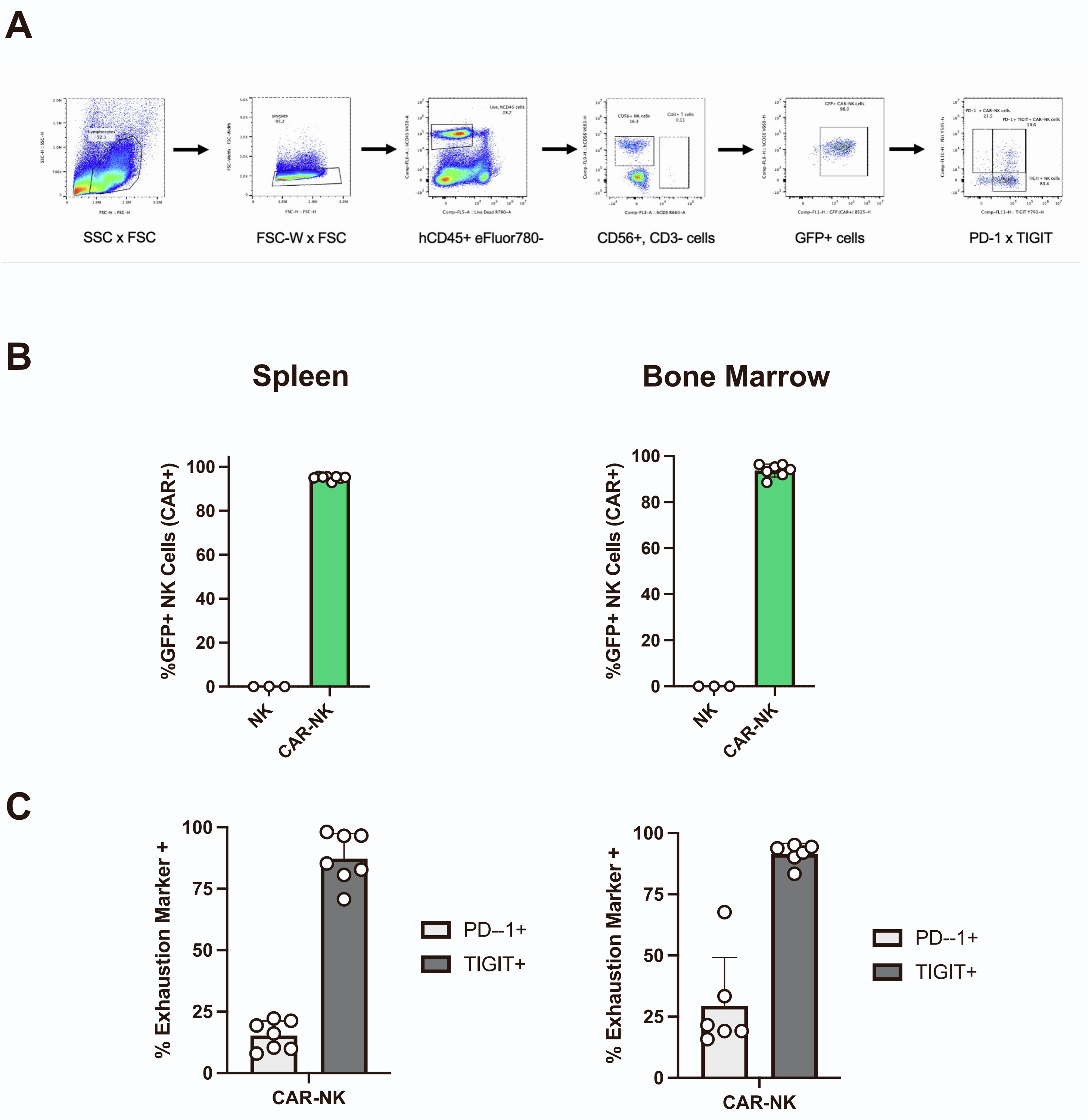

Figure S8 - Remaining NK cells at *in vivo* endpoint show construct retention and expression of CAR construct in both bone marrow and spleen. (A) Gating strategy for spleen and bone marrow CAR-NK cell analysis. **(B)** %CAR-cassette expressing CD56+ cells detected in mice analyzed. **(C)** Exhaustion marker analysis showing %PD-1+ and %TIGIT+ CAR-NK mice with detectable CAR-NK in spleen (left, n=7) and bone marrow (right, n=6).

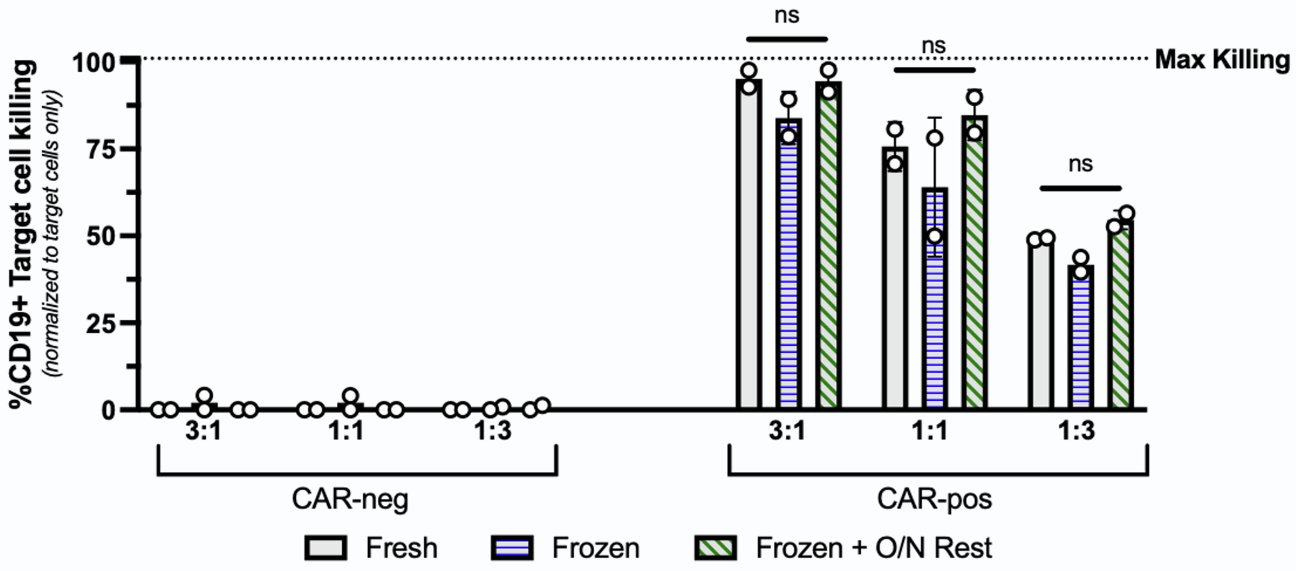

Figure S9. CAR-T killing shows no difference in engineered and cultured or cryopreserved states. Statistical analyses were done using a one-way ANOVA followed by Tukey's multiple comparisons test at each E:T ratios, n = 2 independent biological donors (ns=not significant).

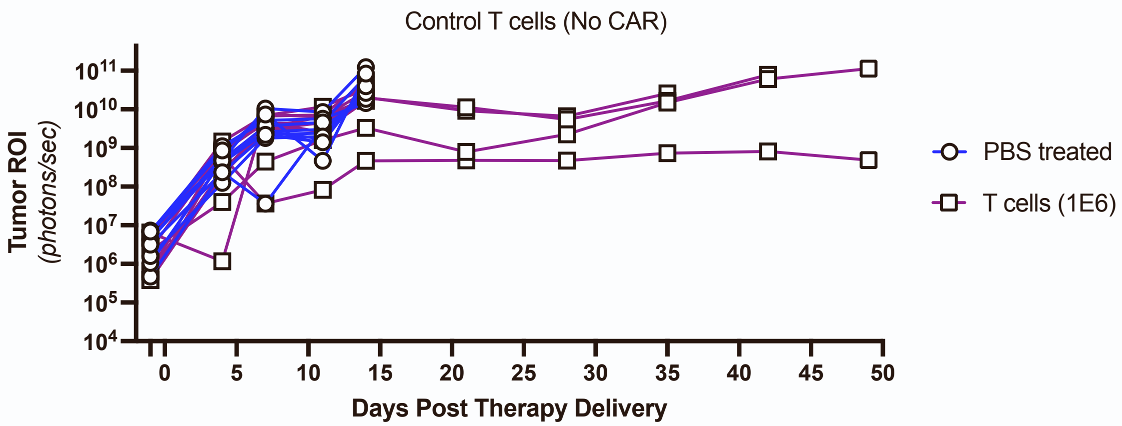

Figure S10 – Individual tumor ROIs from Raji tumor bearing mice receiving control T cell therapy (*1E6 cells, n=10, 2 donors*)**.** Individual tumor ROI values over time.
